# Polysaccharide-Modified Gold Nanorod and Polydopamine Nanocarriers for Near-Infrared Light-Responsive Biomedical Applications: A Comparative Review

**DOI:** 10.3390/molecules31183356

**Published:** 2026-09-21

**Authors:** Yanping Zhang, Yibo Wen

**Affiliations:** College of Chemistry and Chemical Engineering, Henan University of Science and Technology, Luoyang 471000, China; 240320251505@stu.haust.edu.cn

**Keywords:** near-infrared response, gold nanorods, polydopamine, polysaccharide modification, photothermal therapy

## Abstract

Near-infrared (NIR)-responsive nanocarriers offer spatially and temporally controlled heating, drug release, and imaging for biomedical applications. Gold nanorods (AuNRs) and polydopamine (PDA) are two widely investigated platforms with complementary properties: AuNRs provide spectrally tunable plasmonic heating and strong optical imaging capability, whereas PDA offers broadband absorption, adhesive surface chemistry, high cargo-loading capacity, and compatibility with hydrogels and regenerative matrices. Polysaccharide modification can improve physiological stability, reduce nonspecific biological interactions, and introduce receptor targeting, mucoadhesion, or microenvironment responsiveness. This review compares the conjugation chemistry, therapeutic mechanisms, pharmacokinetics, biosafety, and biomedical applications of polysaccharide-modified AuNR and PDA systems, with emphasis on tumor therapy, antibacterial treatment, wound repair, and local drug delivery. AuNR-based platforms are most suitable when rapid wavelength-selective heating and image guidance are primary requirements, whereas PDA-based systems are generally better suited to sustained delivery, tissue adhesion, and long-term local integration. We further discuss AuNR–PDA hybrid architectures and identify key translational barriers, including inconsistent characterization, uncertain long-term fate, nonstandardized photothermal dosimetry, and limited manufacturing reproducibility. This function-oriented comparison provides a practical framework for selecting polysaccharides and carrier platforms according to administration route, biological barrier, therapeutic objective, and safety requirements.

## 1. Introduction

Near-infrared (NIR) light has become an attractive external stimulus for biomedical applications due to its deep tissue penetration and low autofluorescence [1]. While the traditional NIR-I window (700–950 nm) has been extensively explored, recent advances have shifted focus toward the NIR-II (1000–1700 nm) and even NIR-IIa (1300–1400 nm) and NIR-IIb (1500–1700 nm) windows, which offer reduced scattering and higher maximum permissible exposure [2]. AuNRs can be synthetically tuned to absorb in these longer wavelengths, while PDA exhibits broad-band absorption extending into the NIR-II region. Leveraging the thermal effect (photothermal therapy, PTT) or reactive oxygen species (ROS) generation via photodynamic therapy (PDT)—where a photosensitizer absorbs photons of appropriate wavelength (typically 630–900 nm, depending on the photosensitizer’s electronic structure) and transfers energy to molecular oxygen to produce cytotoxic ROS—NIR-responsive nanocarriers have shown great promise for tumor treatment, antibacterial therapy, and controlled drug release [3]. Despite the proliferation of various NIR-responsive nanomaterials—including carbon nanotubes, graphene, black phosphorus, transition metal dichalcogenides (e.g., MoS_2_), and upconversion nanoparticles—AuNRs and PDA stand out as particularly compelling model systems for polysaccharide modification due to their distinct and complementary attributes. Unlike spherical gold nanoparticles, whose single localized surface plasmon resonance (LSPR) band near 520 nm falls outside the optimal biological transparency window, AuNRs possess a longitudinal LSPR mode that can be precisely tuned into the NIR region by adjusting the aspect ratio [4,5]. This NIR-tunable absorption is the primary reason for selecting nanorods, as it ensures deeper tissue penetration and higher photothermal conversion efficiency [6]. In addition, their anisotropic shape affords a larger absorption cross-section, enhanced contrast for photoacoustic imaging (PAI), dark-field microscopy, and computed tomography (CT), and convenient Au–S surface functionalization, making them particularly suitable for theranostic applications [7,8]. PDA, conversely, exhibits broad-band NIR absorption, robust adhesive properties arising from catechol and amine functionalities, and reported biodegradability, which makes it potentially favorable for implantable and injectable formulations; however, its degradation rate and long-term metabolic fate remain incompletely characterized [9,10]. Nevertheless, unmodified AuNRs and PDA tend to aggregate in physiological environments, display poor stability, and lack active targeting, which limits their clinical translation [11,12]. Polysaccharide modification effectively addresses this challenge by improving colloidal stability, reducing nonspecific biological interactions, and introducing active targeting capabilities. This convergence of opportunity and need justifies our focused comparison, while other NIR materials are discussed only where polysaccharide-based functionalization has been explored.

### 1.1. Polysaccharides as Surface Modifiers

Polysaccharides are natural macromolecular carbohydrates composed of monosaccharide units linked by glycosidic bonds [13]. They are abundant in animals, plants, and microorganisms, and are major components of many traditional Chinese medicines [14]. Owing to their favorable biocompatibility, biodegradability, and abundant functional groups, polysaccharides are widely used for surface modification of nanocarriers [15]. Polysaccharide coating not only improves colloidal stability and reduces immune clearance but also enables active targeting via receptor–ligand interactions (e.g., CD44-hyaluronic acid binding) and imparts microenvironment responsiveness (pH, enzyme, or redox) [16].

### 1.2. Publication Landscape and Timeliness

**Figure 1:**
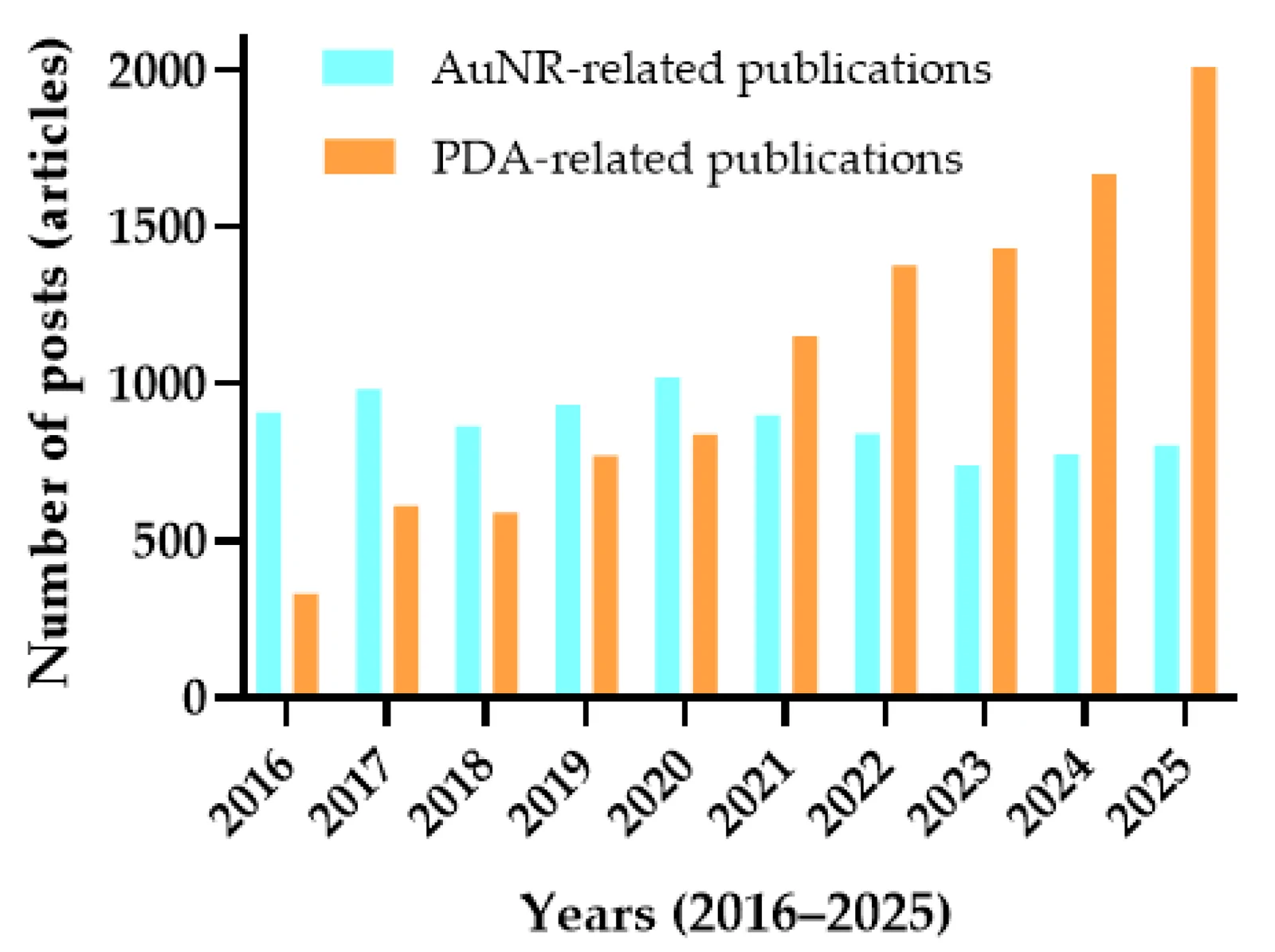
Publication trends of polysaccharide-modified gold nanorods and polydopamine nanocarriers from 2016 to 2025. Data were retrieved from the Web of Science Core Collection using the search terms: (1) “gold nanorods” AND “polysaccharide” AND (“NIR” OR “near-infrared” OR “photothermal”), and (2) “polydopamine” AND “polysaccharide” AND (“NIR” OR “near-infrared” OR “photothermal”). The database was accessed on 8 September 2026.

To provide a macroscopic overview of the research landscape and justify the timeliness of this comparative review, we analysed publication outputs for AuNR- and PDA-related studies over the 2016–2025 period (Figure 1). As shown in the histogram, AuNR-associated publications remained relatively stable across the decade, whereas PDA-related articles exhibited a pronounced continuous increase, surpassing AuNR publication numbers after 2020. This rapidly expanding body of PDA-oriented work, alongside sustained interest in AuNR platforms, highlights the necessity for a comparative review. Moving beyond scattered individual studies, such a review can establish function-driven design guidelines for these two popular nanomaterial systems.

### 1.3. Scope, Literature Selection, and Classification of PDA Systems

For this narrative review, we retrieved literature mainly from the Web of Science Core Collection, PubMed, and Scopus, supplemented by Google Scholar for citation tracking. In addition to the search terms used for Figure 1, keywords included “hyaluronic acid”, “chitosan”, “dextran”, “alginate”, “pullulan”, “chondroitin sulfate”, “heparin”, “photothermal”, “drug delivery”, “wound healing”, “antibacterial”, “tumor therapy”, “pharmacokinetics”, “biosafety”, and “surface modification”. Studies were included if they reported polysaccharide-modified AuNR or PDA systems relevant to NIR-responsive biomedical applications, including synthesis and conjugation chemistry, therapeutic mechanisms, pharmacokinetics, biosafety, and application performance. Non-biomedical applications such as food packaging, environmental remediation, and solar desalination were excluded. To distinguish AuNRs from other gold nanostructures, we focused on anisotropic gold nanorods with a tunable longitudinal LSPR band, and excluded spherical gold nanoparticles, nanoshells, nanostars, and nanocages unless used for mechanistic comparison. For PDA-based systems, a broad range of forms was considered, including nanoparticles, hydrogels, coatings, films, and composites, provided that PDA contributed to NIR-responsive biomedical function or polysaccharide conjugation. However, to avoid the common problem of treating all PDA materials as a single category, we classified them into three functional architecture groups: (i) systemic PDA nanoparticles/nanocarriers, including solid or mesoporous PDA nanoparticles and PDA-coated mesoporous nanoparticles, typically used for intravenous or systemic administration; (ii) local PDA-containing hydrogels or depots, including injectable and bulk hydrogels, fibers, microspheres, and other formulations intended for local sustained release or implantation; (iii) PDA coatings or interface-engineering materials, such as films, scaffolds, membranes, biosensor surfaces, and bioadhesives, where PDA is used mainly as an interfacial modifier rather than a circulating carrier. These categories differ substantially in biodistribution, clearance, drug-release mechanisms, tissue exposure, and safety requirements, and are therefore treated separately in subsequent comparisons. Because this is a narrative review, no formal meta-analysis or risk-of-bias assessment was performed.

### 1.4. Objectives and Structure of This Review

On the basis of the literature landscape and inclusion criteria described above, this review compares two representative material platforms: polysaccharide-modified AuNRs and polysaccharide-functionalized PDA systems. In recent years, two systems—polysaccharide-modified AuNRs and polysaccharide-functionalized PDA systems—have achieved substantial progress in NIR-controlled drug release and synergistic therapy. For example, *Poria cocos* polysaccharide-modified AuNRs (FLP-MPBA-AuNRs) have achieved efficient photothermal anti-tumor effects [17]. Combining PDA nanoparticles with *Bletilla striata* polysaccharide hydrogel triggers CO_2_ release under NIR laser irradiation [18]. This narrative review aims to summarize recent advances in polysaccharide-modified AuNR and PDA nanocarriers, analyze and compare their modification strategies, applications, and development trends, and provide a reference for future research.

## 2. Common Polysaccharide Types and Their Functions

Polysaccharides are widely used to modify NIR light-responsive nanocarriers due to their excellent biocompatibility, biodegradability, easy functional modification, and potential targeting ability, improving their stability, biodistribution, and therapeutic effects.

Hyaluronic acid (HA) is an acidic mucopolysaccharide that lubricates joints, regulates vascular permeability, modulates protein and water–electrolyte diffusion, and promotes wound healing [19,20,21,22,23]. HA can also specifically recognize CD44 receptors overexpressed on the surface of tumor cells to achieve active targeted delivery [24]. However, this targeting strategy is subject to the caveats discussed in Section 7.1, including molecular weight dependence, receptor accessibility, and protein-corona interference. In NIR photothermal therapy, HA-modified AuNRs can be used for pH-dominated and pH/temperature dual-sensitive drug release, achieving synergistic anti-tumor efficacy and deep tumor penetration [25]. HA-modified PDA exhibits excellent NIR photothermal conversion performance, achieving 100% antibacterial activity against *Staphylococcus aureus* and *Escherichia coli* under NIR light irradiation, while the antibacterial effect is limited in the dark, making it a potential photothermal antibacterial material activated by NIR light [26].

Chitosan (CTS) is a cationic polysaccharide with good bioadhesive and mucosal permeation properties. It can form gels, films, fibers, sponges, nanoparticles, nanofibers, and nanocapsules [27,28]. Chitosan can bind heavy metals, dyes, and proteins, and inhibit bacterial and fungal growth [29]. Its amino groups are easily crosslinked or grafted to impart pH-responsive release [30]. Chitosan-modified gold nanocomposites have shown efficient photothermal ablation of liver cancer cells under NIR laser irradiation [31].

Dextran is a neutral bacterial polysaccharide often used as a “stealth” coating. It possesses immunomodulatory, antitumor, anti-inflammatory, antioxidant, hypoglycemic, and wound-healing properties [32,33,34,35]. As a drug carrier, dextran offers high molecular weight, good water solubility, and a modifiable surface, enabling the formation of nanoparticles, nanocapsules, and prodrug complexes that improve drug stability and prolong half-life [36].

Alginate is an anionic polysaccharide extracted from brown algae, known for its mild gelation and biosafety. It is often used to prepare nanogels via ionic crosslinking for encapsulating NIR photosensitizers or chemotherapeutic drugs [37]. Its carboxyl groups become protonated in acidic environments, leading to gel network relaxation and accelerated drug release, suitable for the acidic tumor microenvironment. It is also used to stabilize inorganic photothermal nanoparticles [38].

Pullulan is a neutral polysaccharide produced by black yeast with good film-forming properties and plasticity [39]. Due to the abundance of hydroxyl groups in the molecule, pullulan is prone to chemical modification (e.g., grafting hydrophobic groups, carboxyl groups, or amino groups), thereby constructing self-assembled micelles, nanoparticles, or hydrogels, which are widely used in drug delivery, food packaging, cosmetic substrates, and functional modification of NIR light-responsive nanocarriers, combining biodegradability and good physiological compatibility [40].

Chondroitin sulfate (ChS) is a sulfated glycosaminoglycan present in connective tissues such as cartilage, conferring compressive resistance and participating in cell signaling [41]. Due to its good biocompatibility, degradability, and negative charge, it is widely used in joint health, anti-inflammation, and tissue engineering [42]. In NIR-responsive nanocarriers, its negative charge interacts with cationic photothermal materials (e.g., AuNRs, black phosphorus) to achieve stable dispersion, prolonged circulation, and targeting or pH/enzyme-responsive drug release [43].

Heparin is a highly sulfated glycosaminoglycan with high negative charge density, enabling it to bind tightly to heparin-binding proteins such as VEGF and FGF [44]. It stabilizes growth factors, protects them from enzymatic degradation, and possesses intrinsic anti-inflammatory, anticoagulant, and pro-angiogenic properties [45]. Heparin can be integrated into microspheres, nanofibers, hydrogels, and other platforms via electrostatic adsorption, physical blending, covalent grafting, and network crosslinking to achieve spatiotemporally controlled release of growth factors, with applications in soft tissue regeneration, osteochondral repair, nerve repair, cardiovascular regeneration, wound healing, anti-fibrotic therapy, and tumor microenvironment modulation [46].

*Poria cocos* polysaccharide is a β-glucan extracted from the sclerotium of *Wolfiporia cocos*, a medicinal fungus widely used in traditional Chinese medicine. It exhibits immunomodulatory and antitumor activities through activation of macrophages, dendritic cells, and natural killer (NK) cells, as well as promotion of cytokine secretion [17]. In NIR-responsive nanocarrier systems, *Poria cocos* polysaccharide has been successfully conjugated to AuNRs via MPBA-mediated boronate ester linkages, achieving combined photothermal ablation and immune activation for enhanced antitumor efficacy and reduced tumor recurrence [17].

*Bletilla striata* polysaccharide is a glucomannan extracted from the orchid *Bletilla striata*. It possesses favorable biocompatibility, hemostatic activity, and wound-healing promotion properties, attributed to its ability to accelerate granulation tissue formation, collagen deposition, and re-epithelialization [18]. In PDA-based systems, *Bletilla striata* polysaccharide hydrogel integrated with PDA nanoparticles has been designed for NIR-triggered CO_2_ release, which alleviates hypoxia in diabetic wounds through the Bohr effect, promoting angiogenesis and tissue regeneration [18].

*Belamcanda chinensis* polysaccharide is extracted from the rhizome of *Belamcanda chinensis* (L.) DC., a medicinal plant used in traditional Chinese medicine. It is primarily composed of mannose, glucose, and galactose in varying molar ratios, with some fractions also containing galacturonic acid and arabinose [47,48]. This polysaccharide exhibits multiple pharmacological activities, including antitumor, antioxidant, and immunomodulatory effects. In NIR-responsive nanocarrier systems, *Belamcanda chinensis* polysaccharide has been conjugated to AuNRs for photothermal cancer therapy [47].

Astragalus polysaccharide is derived from the root of *Astragalus membranaceus*, a leguminous plant widely used in traditional medicine. It possesses well-documented immunostimulatory properties, including promotion of dendritic cell maturation, macrophage polarization toward the M1 phenotype, and T-cell proliferation [49]. In photothermal immunotherapy systems, Astragalus polysaccharide has been encapsulated with AuNRs in PEGylated PLGA nanoparticles, where it synergistically enhances antitumor immune responses when combined with focused ultrasound and NIR irradiation [50].

Glycyrrhiza polysaccharide is extracted from the root of *Glycyrrhiza uralensis* (licorice). It exhibits immunomodulatory activities, including enhancement of antigen-specific immune responses and promotion of dendritic cell maturation [51]. When conjugated with gold nanoparticles, Glycyrrhiza polysaccharide has been used as an HPV-DC vaccine adjuvant, promoting T-cell proliferation and antitumor immunity [51].

Porphyran is a sulfated galactan polysaccharide extracted from red algae of the genus *Porphyra*. It possesses antioxidant, immunomodulatory, and antitumor activities [52]. In NIR-responsive systems, pH-responsive porphyran-coated AuNRs have been developed for combined photothermal therapy and immunotherapy, where the polysaccharide shell not only improves colloidal stability but also contributes to dendritic cell maturation and immune activation [52].

*Pleurotus ferulae* polysaccharide is a fungal β-glucan derived from the edible mushroom *Pleurotus ferulae*. It has been shown to enhance dendritic cell maturation, promote antigen-specific immune responses, and inhibit tumor growth in preclinical models [53]. Gold nanoparticles functionalized with *Pleurotus ferulae* polysaccharide have demonstrated potent immunomodulatory effects and antitumor activity [53]. The main characteristics, targeting mechanisms, and representative applications of these common polysaccharides in AuNR and PDA nanocarrier systems are summarized in Table 1 for rapid cross-comparison. 

Taken together, polysaccharide selection should be guided by the intended administration route, the principal biological barrier, and the desired carrier function rather than by material availability alone. For example, hyaluronic acid may be prioritized for targeting tumors with elevated CD44 expression, although its performance depends strongly on molecular weight, receptor accessibility, and the stability of surface presentation. Neutral and highly hydrophilic polysaccharides, such as dextran, can serve as potential stealth coatings for intravenously administered nanocarriers when prolonged circulation and reduced nonspecific protein adsorption are required. In contrast, chitosan is particularly attractive for oral, nasal, and other mucosal delivery routes because of its mucoadhesive properties and ability to transiently enhance epithelial permeability. Anionic polysaccharides, including alginate, may be preferred when ionic complexation, pH-responsive behavior, or protection of sensitive payloads in the gastrointestinal tract is required. Thus, a practical selection sequence should consider: (i) the route of administration and target tissue, (ii) the dominant extracellular and intracellular barriers, (iii) the required functions, such as targeting, stealth, mucoadhesion, or stimulus responsiveness, and (iv) material-specific variables, including molecular weight, charge density, degree of substitution, degradability, and batch reproducibility. This function-oriented framework represents an essential first step toward the rational and precise design of polysaccharide-based nanomedicines.

To facilitate the practical application of this framework, the key decision variables are summarized in Table 2, which outlines a stepwise pathway from administration route to main safety constraint. The table is intended as a guide rather than a rigid prescription; final selection should also consider molecular weight, charge density, degree of substitution, and batch reproducibility.

## 3. Polysaccharide Modification Strategies and Chemistry

**Figure 2:**
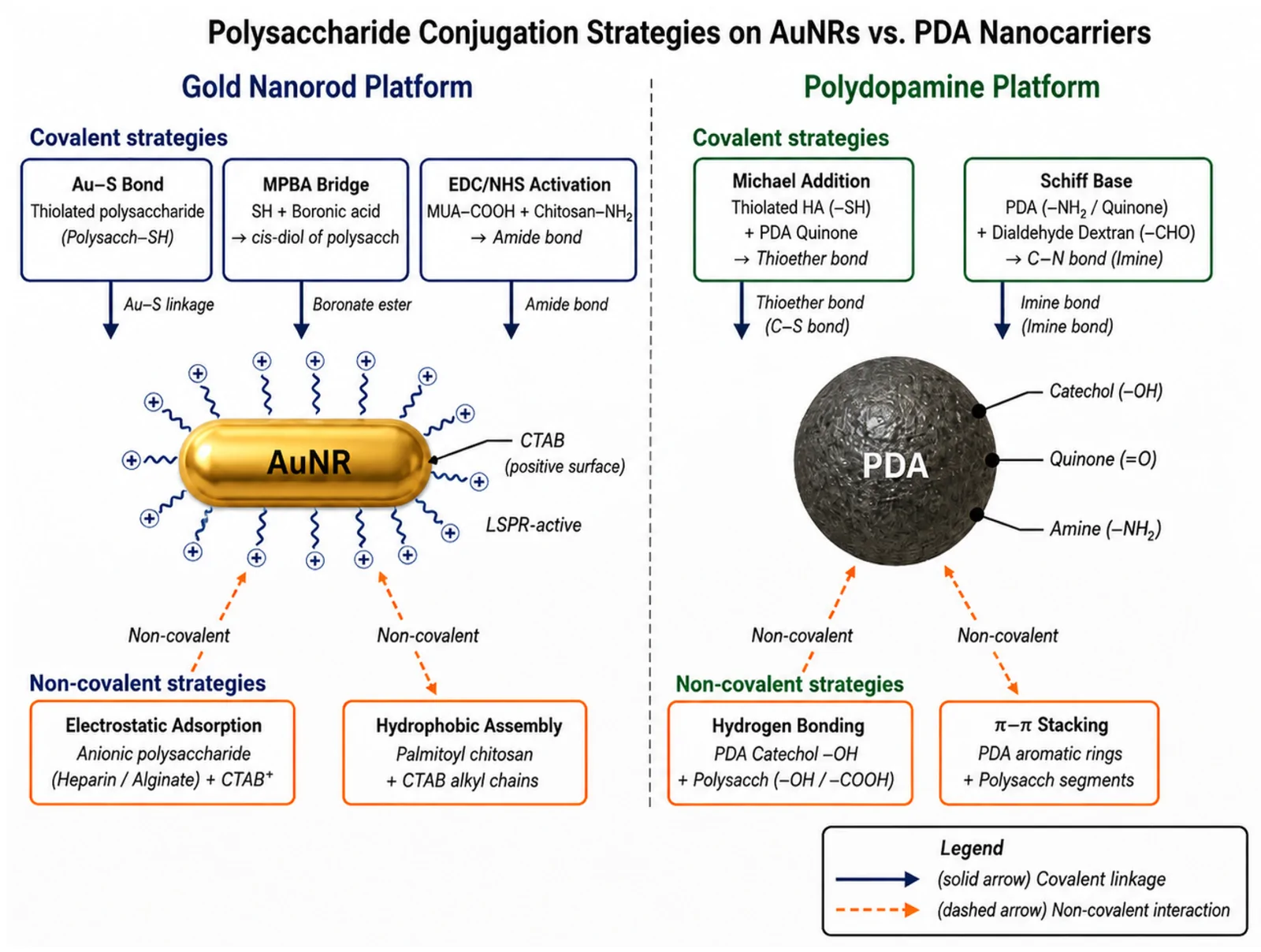
Schematic comparison of polysaccharide conjugation chemistries on AuNR and PDA platforms. Covalent and non-covalent strategies are categorized for each carrier, with key functional groups and bond types annotated. Abbreviations: AuNR, gold nanorod; PDA, polydopamine; MPBA, 4-mercaptophenylboronic acid; MUA, 11-mercaptoundecanoic acid; EDC, 1-ethyl-3-(3-dimethylaminopropyl)carbodiimide; NHS, N-hydroxysuccinimide; HA, hyaluronic acid.

Before detailing the specific conjugation methods for each carrier, Figure 2 provides a visual overview of the covalent and non-covalent polysaccharide functionalization strategies on both AuNR and PDA platforms, highlighting the key functional groups and bond types involved in each approach.

### 3.1. Conjugation Methods for Polysaccharides with Gold Nanorods

Polysaccharides can be conjugated to gold nanorods (AuNRs) via either covalent bonding or non-covalent interactions. Covalent strategies provide robust and stable linkages that enhance the circulation time and structural integrity of the nanocomposite. Thiolated polysaccharides are anchored onto the gold surface via Au–S bonds. Zhao et al. utilized 4-mercaptophenylboronic acid (MPBA) as a bridging molecule to immobilize *Poria cocos* polysaccharide onto AuNRs, where the thiol group of MPBA anchors to the gold surface via Au–S bonds and the boronic acid group forms boronate ester linkages with the polysaccharide’s cis-diols [17,47]. Alternatively, a ligand exchange step with 11-mercaptoundecanoic acid (MUA) introduces carboxyl groups onto the AuNR surface, which can be activated by EDC/NHS to form amide bonds with the amino groups of chitosan [72]. Nguyen et al. used glucosamine as both a stabilizer and a reducing agent; upon heating, the amino groups of glucosamine reduced Au^3+^ to Au^0^, forming gold nanoparticles simultaneously encapsulated within cross-linked glucosamine/alginate polysaccharide networks [73].

Non-covalent interactions, encompassing electrostatic adsorption, hydrophobic assembly, and physical entrapment, offer operational simplicity and generally preserve the native conformation of polysaccharides. As reviewed by Pissuwan & Niidome (2015), negatively charged polyelectrolytes can coat positively charged cetyltrimethylammonium bromide (CTAB)-coated AuNRs via electrostatic attraction, a principle applicable to polysaccharide coatings [65]. Negatively charged heparin can coat positively charged CTAB-coated AuNRs via electrostatic attraction [74]. de Barros et al. demonstrated that sulfated polysaccharides can efficiently interact with the surface of CTAB-coated AuNRs through their sulfate and carboxyl groups. Specifically, sulfated chitosan produced well-dispersed individual AuNRs wrapped by a dense polysaccharide layer, whereas gum arabic led to nanoparticle clustering, as evidenced by UV–vis, FTIR, and electron microscopy [66].

### 3.2. Conjugation Methods for Polysaccharides with Polydopamine

Polydopamine, rich in catechol and amine groups, interacts with polysaccharides through physical adsorption or chemical bonding. Physical adsorption and co-deposition exploit the strong adhesion of PDA. Wang et al. directly added PDA nanoparticles to an oxidatively crosslinked *Bletilla striata* polysaccharide hydrogel precursor; the strong adhesion led to deposition on the polysaccharide chain surface, forming a “polysaccharide core–PDA shell” structure [18]. Kolgesiz et al. achieved conjugation between PDA and pectin via hydrogen bonding and π–π stacking, followed by Ca^2+^ ion crosslinking to stabilize the hybrid hydrogel network [75].

Covalent coupling is the most robust strategy. Yu et al. chemically conjugated PDA with thiol-functionalized hyaluronic acid via Michael addition, constructing a composite hydrogel with both biocompatibility and sustained drug release [68]. Chu et al. used carboxymethyl chitosan (CMCS) and dialdehyde dextran (DHG) to form Schiff base bonds; the amino groups of the inner PDA layer further formed Schiff base bonds with aldehyde groups of DHG, creating a dual-protection carrier [76].

## 4. Biomedical Applications of Polysaccharide-Modified AuNRs

**Figure 3:**
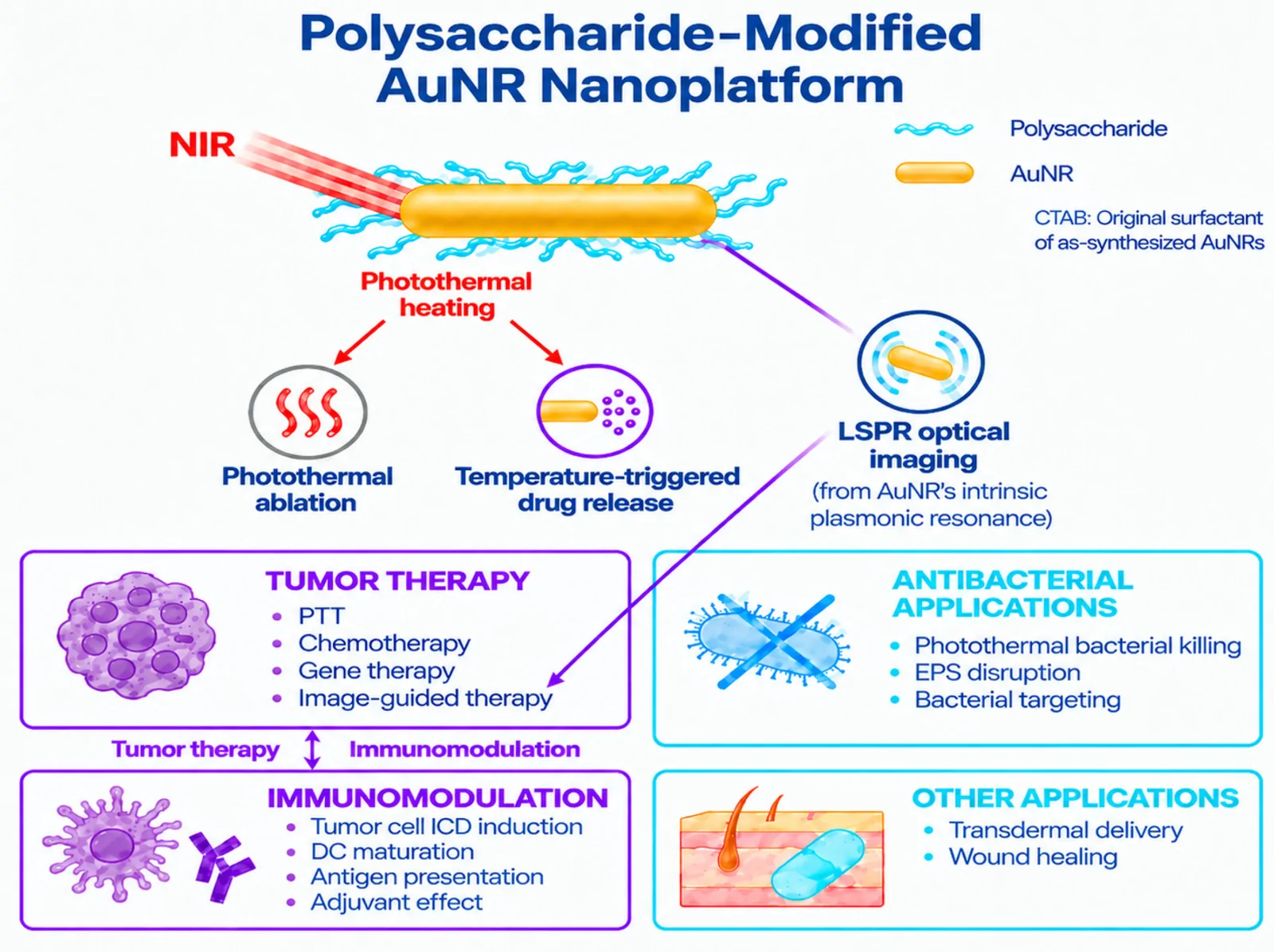
Schematic illustration of polysaccharide-modified AuNR nanoplatforms for NIR-responsive biomedical applications. The core nanostructure (AuNR core with a polysaccharide shell replacing CTAB) generates photothermal heating and LSPR optical imaging upon NIR irradiation. These functional outputs support four major biomedical application areas: tumor therapy, immunomodulation, antibacterial treatment, and other applications (transdermal delivery and wound healing). Abbreviations: AuNR, gold nanorod; NIR, near-infrared; CTAB, cetyltrimethylammonium bromide; LSPR, localized surface plasmon resonance; PTT, photothermal therapy; DC, dendritic cell; EPS, extracellular polymeric substance.

Polysaccharide modification expands the biomedical utility of AuNRs by improving colloidal stability, attenuating CTAB-associated toxicity, and introducing targeting or stimulus-responsive functions. The principal biomedical applications are summarized in Figure 3 and discussed below.

### 4.1. Tumor Therapy

Solid tumors present major challenges for nanodrug delivery due to their heterogeneous microenvironment and dense extracellular matrix (ECM) [77]. Conventional photothermal nanomaterials often suffer from insufficient tumor accumulation and poor deep penetration. The tunable LSPR of AuNRs enables strong absorption within biologically relevant NIR windows, while polysaccharide modification facilitates tumor targeting and microenvironment-responsive delivery.

PTT uses photothermal materials to convert NIR light into heat, killing cancer cells [78]. Polysaccharide modification improves AuNR dispersion and circulation, enabling efficient tumor accumulation. In general, mild hyperthermia (approximately 41–45 °C) has been associated with apoptotic cell death, whereas higher temperatures (above ~50 °C) are generally linked to coagulative necrosis. However, these values should be regarded as approximate, context-dependent ranges rather than strict thresholds. The actual biological outcome—apoptosis, necrosis, immunogenic cell death (ICD), or vascular damage—depends not only on the peak temperature but also on the duration of heating, heating rate, tissue type, perfusion, and the spatial temperature distribution within the tumor. Consequently, thermal dose, commonly expressed as cumulative equivalent minutes at 43 °C (CEM43), provides a more reliable predictor of tissue response than peak temperature alone [79,80]. Paola et al. coated AuNRs with lipoic acid and gellan gum; NIR laser-mediated PTT destroyed HCT116 colon cancer cells with a 75% killing rate [81]. Zhou et al. replaced CTAB with thiolated lemon polysaccharide (SH-CL90) to prepare AuNRs-SH-CL90, which showed a photothermal conversion efficiency of 22.2% and inhibited proliferation of HepG2, A549, and MCF-7 cells [82]. Zhao et al. used *Belamcanda chinensis* polysaccharide-modified AuNRs for targeted liver cancer therapy, inducing apoptosis, cell cycle arrest, and anti-angiogenesis, with low toxicity in zebrafish [47].

Gene–photothermal combination therapy uses physical heat to complement gene regulation. Cationic polysaccharides can encapsulate negatively charged nucleic acids [83]. Dai et al. constructed a Janus chitosan–gold nanohybrid (J-ACP) for photoacoustic imaging-guided synergistic gene/photothermal therapy, significantly inhibiting breast cancer growth [84]. Sun et al. designed MMP-2-responsive AuNRs loaded with HSP-70 siRNA to reduce thermotolerance and enhance PTT [85].

Chemotherapy–photothermal combination therapy co-delivers chemotherapeutic agents and photothermal materials. Xu et al. developed an HA- and hydroxyethyl chitosan-modified AuNR platform with charge-reversal and dual-responsive drug release, achieving superior combined efficacy against MCF-7 cells [86].

Photothermal therapy–immunotherapy uses PTT to release tumor antigens and activate systemic antitumor immunity [87]. Xiong et al. encapsulated Astragalus polysaccharide and AuNRs in PEGylated PLGA nanoparticles; combined with focused ultrasound, the system showed significant antitumor effects and immune activation [50]. Zhao et al. used *Poria cocos* polysaccharide–AuNR FLP-MPBA-AuNRs to achieve photothermal ablation and immune activation, reducing recurrence and metastasis [17].

Collectively, these studies show that the principal advantage of polysaccharide-modified AuNRs is not merely efficient heat generation, but the integration of a spectrally defined photothermal core with biologically active interfaces. Polysaccharides play three distinct roles: they replace or shield cytotoxic CTAB, promote receptor-mediated accumulation, and provide drug- or gene-binding domains for combination therapy. Among the therapeutic configurations discussed above, gene–PTT combinations are particularly rational because silencing heat-shock proteins directly addresses an adaptive mechanism of thermoresistance, whereas chemotherapy–PTT systems mainly improve local drug release and membrane permeability. Immunotherapy combinations offer the greatest potential for controlling distant lesions and recurrence, but they also introduce greater variability because immune activation depends on thermal dose, tumor immunogenicity, and the intrinsic activity of the selected polysaccharide.

The limitations of this platform should also be distinguished from its strengths. AuNRs generate rapid and spatially confined heating at the LSPR wavelength, which is advantageous for image-guided tumor ablation, but their drug-loading capacity is generally lower than that of mesoporous PDA, and their non-degradable gold core may pose a potential concern for repeated systemic administration, although direct evidence for this limitation is currently limited. Moreover, enhanced uptake by polysaccharide receptors does not necessarily guarantee deep tumor penetration, because receptor-mediated internalization may trap nanoparticles in perivascular cells. Thus, AuNR-based systems are most compelling when rapid photothermal ablation and imaging are the primary objectives, whereas sustained multidrug delivery or repeated local treatment may favor PDA-based formulations. Future studies should therefore report not only tumor-volume inhibition but also intratumoral spatial distribution, thermal-dose maps, recurrence rates, and long-term gold retention.

### 4.2. Antibacterial and Antibiofilm Applications

Biofilms are bacterial communities encased in extracellular polymeric substances, causing chronic infections and antibiotic resistance [88,89]. Polysaccharide-modified AuNRs combat biofilms through “photothermal killing–targeted enrichment–biofilm degradation.” He et al. used fucose-containing polymer-modified AuNRs with α-amylase to degrade biofilm EPS, achieving nearly 100% killing of planktonic bacteria and 70.9% biofilm biomass reduction [67]. Lv et al. developed lipopolysaccharide aptamer-functionalized MoS2-coated AuNRs; 5 min of NIR irradiation raised the temperature by 13 °C, achieving 100% killing of multidrug-resistant *P. aeruginosa* and 70% biofilm disruption [90]. Jiménez et al. embedded AuNRs in a chitosan hydrogel; 2 min of irradiation (10 °C rise) significantly killed bacteria without cytotoxicity, suggesting potential for periodontitis treatment [91].

These examples indicate that successful antibiofilm treatment requires more than an increase in temperature. The dense extracellular polymeric substance matrix limits nanoparticle diffusion and produces spatially heterogeneous heating; accordingly, the most effective AuNR systems combine photothermal killing with a second function that disrupts the biofilm matrix or promotes bacterial recognition. Enzyme-assisted degradation and aptamer-mediated targeting therefore provide mechanistic advantages over non-targeted heating alone. Polysaccharide hydrogels further localize AuNRs at the infected site and reduce systemic exposure, although excessive crosslinking may impede heat transfer and nanoparticle–biofilm contact.

Compared with PDA-based wound dressings, AuNR formulations are particularly effective when rapid and intense bacterial ablation is required [67,92]. For instance, PDA-based photothermal systems typically require 10 min of NIR irradiation to achieve >99% bacterial killing, whereas AuNR-based systems have demonstrated rapid and near-complete bacterial eradication within minutes of NIR irradiation [67,92,93,94]. Although several AuNR systems achieved substantial bacterial reduction within a few minutes, direct kinetic comparison with PDA systems is currently not possible because the reported studies used different wavelengths, power densities, material concentrations, bacterial species, biofilm models, and endpoint definitions. These studies suggest a potential for rapid AuNR-mediated heating, but do not establish a universal kinetic superiority over PDA [7,95]. This makes AuNRs particularly suitable for acute infection scenarios where immediate pathogen clearance is critical. However, AuNRs contribute relatively little intrinsic antioxidant activity or extracellular-matrix mimicry after bacterial clearance. PDA composites, in contrast, can combine moderate photothermal action with radical scavenging, tissue adhesion, and sustained antibiotic release [96]. This difference suggests a scenario-based choice: AuNRs are more suitable for short-duration ablation of localized, recalcitrant biofilms, whereas PDA hydrogels may be preferable when infection control must be integrated with prolonged tissue repair [96,97].

### 4.3. Immunomodulation and Vaccine Adjuvants

Polysaccharide-modified AuNRs can act as vaccine adjuvants, combining photothermal effects with immunomodulatory activity [98]. Lin et al. developed a pH-responsive porphyran-coated AuNR nanodrug that induced PTT and dendritic cell maturation [52]. Yuan et al. synthesized *Pleurotus ferulae* polysaccharide–AuNPs that enhanced DC maturation and antigen-specific immune responses, inhibiting TC-1 tumor growth [53]. Zhao et al. used glycyrrhiza polysaccharide–AuNPs as an HPV-DC vaccine adjuvant, promoting DC maturation, T-cell proliferation, and strong antitumor immunity [51]. Although these studies used spherical AuNPs rather than AuNRs, they provide relevant evidence regarding the immunomodulatory role of polysaccharide–gold interfaces; they should not, however, be interpreted as direct evidence for AuNR photothermal performance. The immunological contribution of these systems arises from two separable components: photothermal injury can promote ICD and antigen release, whereas bioactive polysaccharides can enhance dendritic-cell maturation or innate immune signaling. This division is important because stronger heating does not necessarily produce a stronger antitumor immune response. Excessive temperatures may cause rapid coagulative necrosis and vascular shutdown, thereby limiting immune-cell infiltration, while controlled hyperthermia may better preserve antigenicity and danger-associated molecular patterns. Consequently, thermal dose, antigen presentation, and systemic cytokine profiles should be evaluated together rather than treating tumor shrinkage as a surrogate for immune activation.

Compared with PDA, AuNRs offer more precise wavelength-dependent heating and stronger photoacoustic or scattering-based imaging, facilitating image-guided immunothermal therapy [8]. Nevertheless, the persistent gold core may potentially complicate repeated vaccination schedules owing to concerns about long-term gold retention, although direct evidence of clinical significance remains limited [99]. PDA may be advantageous for repeated or depot-based immunotherapy because of its degradability and higher cargo-loading capacity, although its antioxidant character could theoretically attenuate ROS-dependent immunogenic signaling under some conditions [100,101]. Direct comparative studies are needed to determine how carrier composition influences the balance between inflammatory activation and tissue protection.

### 4.4. Transdermal Delivery and Oral Administration

Haine et al. used an AuNR-coated polysaccharide hydrogel patch for transdermal protein delivery; NIR irradiation raised skin temperature to 43 °C, disrupting the stratum corneum and enhancing protein penetration. Notably, Haine et al. did not directly characterize protein thermostability under their 43 °C photothermal condition; although ovalbumin’s melting temperature is substantially higher than 43 °C, only FITC fluorescence was used to track protein penetration rather than assays for native protein structure [102]. Kumari et al. developed modified apple polysaccharide–AuNPs (spherical gold nanoparticles) for oral insulin delivery, significantly reducing blood glucose in diabetic rats [103]. These results support the potential of polysaccharide–gold systems for oral delivery but are not direct evidence for AuNR-based delivery.

These non-oncological applications demonstrate that the value of AuNRs extends beyond cytotoxic photothermal therapy. In transdermal delivery, for example, moderate and reversible heating is more important than maximal temperature elevation, because the objective is to transiently increase tissue permeability rather than induce ablation. Similarly, oral protein delivery requires protection from gastrointestinal degradation and preservation of biological activity; here the polysaccharide shell is functionally dominant during transit, whereas the gold core acts as the NIR-triggered photothermal transducer that enables controlled release at the target site [103]. Such applications may offer clearer translational advantages than systemic tumor therapy because the carrier can be administered locally, the irradiation site is accessible, and the required thermal dose is lower. However, they also require application-specific safety endpoints, including skin barrier recovery, protein structural integrity, and nanoparticle leakage from the local formulation.

Overall, polysaccharide-modified AuNRs are best suited to applications requiring rapid, wavelength-selective heating and simultaneous optical imaging. Their strongest evidence remains in tumor ablation and localized antibiofilm therapy, particularly when polysaccharides add receptor targeting or biofilm-disrupting functions. Their main weaknesses—limited loading capacity, potential shape instability under high-power irradiation, and persistent gold retention—may become more consequential in repeated or chronic treatment, although the clinical significance of these concerns remains to be fully established. These limitations provide a clear transition to PDA-based systems, whose broader chemical functionality and reported biodegradability favor sustained delivery and regenerative applications.

## 5. Biomedical Applications of Polysaccharide-Modified PDA

**Figure 4:**
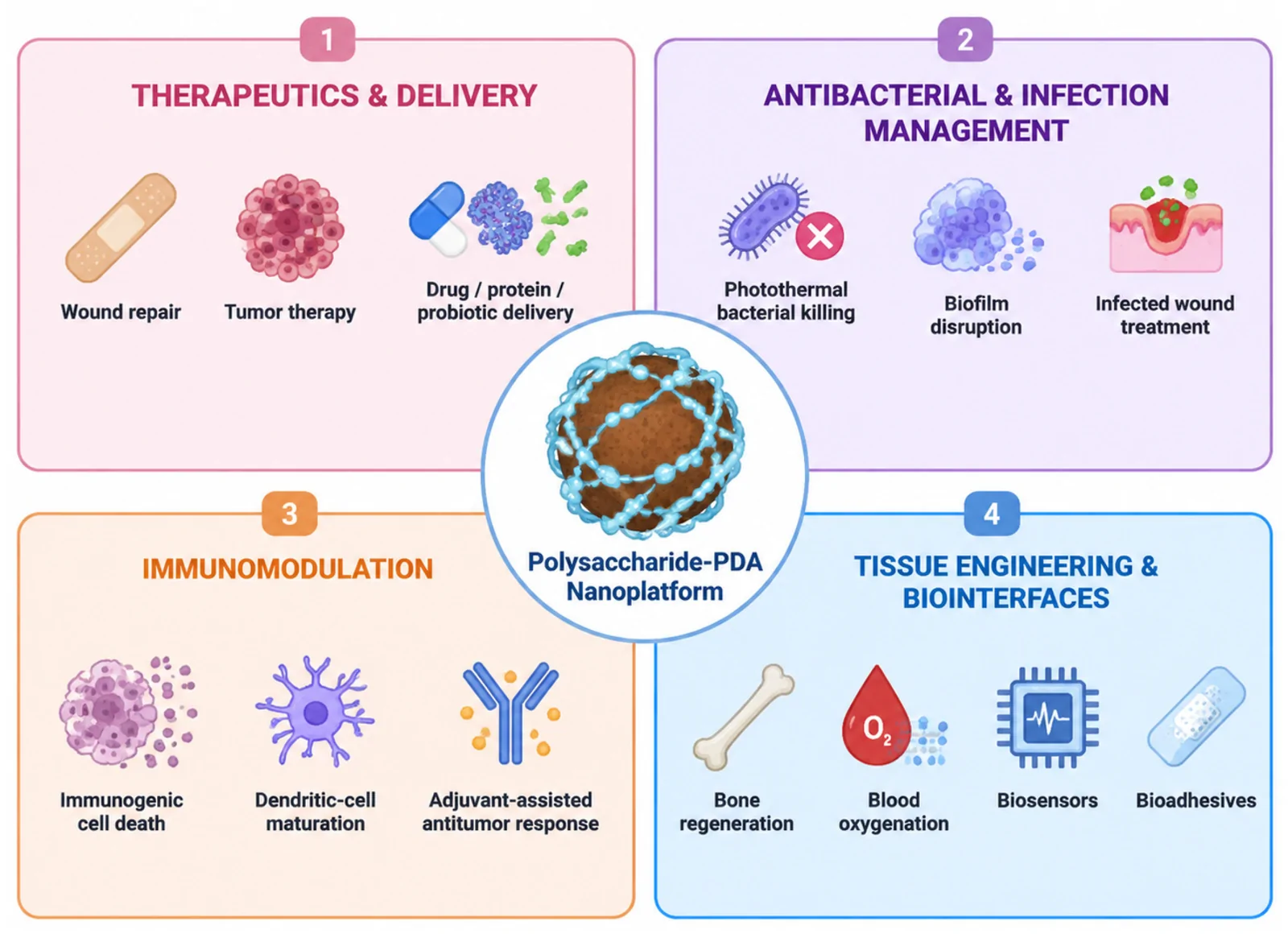
Icon-based overview of the polysaccharide-modified PDA nanoplatform for near-infrared-responsive biomedical applications. The central core represents the hybrid polysaccharide–PDA nanoplatform. Four functional quadrants summarize the main biomedical applications: (1) therapeutics and delivery, including wound repair, tumor therapy, and drug/protein/probiotic delivery; (2) antibacterial and infection management, including photothermal bacterial killing, biofilm disruption, and infected wound treatment; (3) immunomodulation, including immunogenic cell death, dendritic-cell maturation, and adjuvant-assisted antitumor response; and (4) tissue engineering and biointerfaces, including bone regeneration, blood oxygenation, biosensors, and bioadhesives. Abbreviations: PDA, polydopamine.

Polysaccharide-modified PDA nanocarriers integrate the bioadaptability of polysaccharides with the multifunctional properties of PDA, overcoming the limitations of single carriers and enabling biomedical applications such as wound repair, tumor therapy, drug/protein/probiotic delivery, tissue engineering, and biosensing. The biomedical application fields of polysaccharide-modified polydopamine nanocarriers are illustrated in Figure 4.

### 5.1. Wound Repair

The PDA systems discussed in this section are predominantly local PDA-containing hydrogels/depots, with some examples of PDA-containing fibers and microcapsules. They are intended for local application rather than systemic circulation. Unless otherwise stated, their performance should not be directly compared with that of systemically injected PDA nanoparticles. Xu et al. prepared a carboxymethyl chitosan–PDA–quercetin hydrogel in which PDA served as both a photothermal agent and a quercetin carrier, achieving >99% antibacterial activity against *S. aureus* and *E. coli* under NIR and accelerating wound healing [62]. Ghazanfaripanah et al. prepared a chitosan–PDA–graphitic carbon nitride hydrogel in which PDA enhanced photothermal conversion and provided adhesive crosslinking, promoting infected wound healing in mice [104]. Ma et al. developed carboxymethyl *Bletilla striata* polysaccharide–PVA fibers loaded with PDA@metformin microcapsules, where PDA acted as a photothermal trigger and a drug-binding depot, enabling pH-responsive release and NIR-synergized antibacterial effects [105]. Fiorica et al. used gellan gum–PDA hydrogels loaded with ciprofloxacin, in which PDA provided photothermal potentiation of antibiotic activity under NIR irradiation [106]. Zhang et al. prepared dextran–PDA hydrogels in which PDA served as a drug-binding matrix for sustained chlorhexidine release with significant antibacterial activity [107]. O’Connor et al. reported a dextran–PEI-PDA antioxidant hydrogel that promoted cell migration [108].

For diabetic wounds, Wang et al. designed a *Bletilla striata* polysaccharide hydrogel with PDA nanoparticles that, acting as a photothermal trigger for CO_2_ release, modulated the diabetic wound microenvironment and promoted healing [18]. Mechanistically, the photothermal effect of PDA elevates the local temperature, promoting the decomposition of bicarbonate into CO_2_ within the wound microenvironment [18]. The released CO_2_ alleviates hypoxia through two complementary pathways: the Bohr effect, which enhances oxygen delivery by facilitating hemoglobin O_2_ dissociation, and CO_2_-mediated vasodilation and angiogenesis, which improve local perfusion and tissue oxygenation [18]. This CO_2_-generating system synergistically accelerates diabetic wound healing. For frostbite, Cui et al. developed a pullulan-based PDA organohydrogel with anti-freezing and photothermal properties, accelerating frostbite healing [109]. Su et al. prepared agarose–PDA hydrogel scaffolds that promoted full-thickness skin defect healing [110].

Taken together, these studies reveal that PDA contributes to wound healing through a combination of functions rather than through photothermal antibacterial action alone. Its catechol- and quinone-rich surface enhances tissue adhesion and interacts with polysaccharide networks, while its radical-scavenging capacity can reduce excessive oxidative stress during the inflammatory phase [108,110]. PDA also provides binding sites for antibiotics, polyphenols, and metabolic drugs, allowing sustained local release [62,105,107]. The polysaccharide matrix, in turn, maintains a moist environment, absorbs exudate, and provides a provisional extracellular-matrix-like scaffold [62,104,109,110]. Therapeutic performance therefore arises from the division of labor between PDA and the polysaccharide network.

Compared with AuNRs, whose biomedical use is concentrated mainly on rapid tumor or biofilm ablation, the broad application of PDA in wound repair highlights its superior adhesiveness, biodegradability, antioxidant activity, and biomimetic matrix compatibility. These characteristics complement the broadband, wavelength-dependent photothermal behavior of PDA, whose peak efficiency can overlap with that of AuNRs under certain irradiation conditions, although direct comparisons are complicated by differences in measurement protocols, particle size, and irradiation parameters. Importantly, however, antioxidant activity is not universally beneficial: excessive ROS scavenging during the early antimicrobial phase may weaken host defense, whereas insufficient scavenging may prolong inflammation. Future PDA dressings should therefore be designed for stage-adaptive regulation, with early antibacterial heating followed by controlled antioxidant and regenerative activity. Comparative studies should report bacterial burden, inflammatory-cell infiltration, collagen organization, angiogenesis, and scar quality rather than wound-closure rate alone.

### 5.2. Disease Treatment and Drug Delivery

In terms of tumor therapy, Zhu et al. developed a hydrogel composed of chitosan oligosaccharides, oxidized herb polysaccharide and mesoporous PDA, which had injectability, self-healing properties and pH–photothermal dual responsiveness. Through the synergistic effect of photothermal therapy and chemotherapy and anti-inflammatory effects, it showed significant anti-tumor activity against B16 melanoma in vitro and in vivo, with good biocompatibility and low systemic toxicity [111]. In the local treatment of acne, Ain et al. encapsulated Glycyrrhiza glabra polysaccharide (GGP) with mesoporous silica nanoparticles (MSNs) and then surface-modified with PDA. The in vitro release rate reached 89% in an acne site pH 5 environment, which could significantly enhance sebocyte uptake and exert anti-acne effects by inhibiting pro-inflammatory cytokines and related pathways [112].

In the field of protein drug delivery, Han et al. incorporated PDA into the cross-linked network formed by oxidized HA and carboxymethyl chitosan to prepare a PDA NPs@OHA-l-CEC hydrogel, which could achieve continuous sequential release of VEGF (rapid release driven by Fickian diffusion) and BSA (slow release by super Case II transport mechanism), and the cross-linking density and protein release rate could be controlled by adjusting the oxidation degree of OHA [113].

In probiotic delivery, Chu et al. constructed a CMCS-DHG/PDA bilayer carrier composed of carboxymethyl chitosan (CMCS), dialdehyde dextran (DHG) and PDA to form a nano-coating around *Bacillus subtilis*, which had significant pH responsiveness, could protect probiotics from the gastrointestinal environment, improve their tolerance to bile and antibiotics, enhance mucosal adhesion, and improve oral bioavailability and intestinal colonization rate [76].

In antibiotic delivery, Michalicha et al. used chitosan hydrogel as the base material, modified with PDA to improve the binding ability to bioactive molecules containing free amino groups. Taking gentamicin (antibiotic) as a model molecule, it continuously inhibited the growth of pathogenic bacteria such as *Staphylococcus aureus*, *Staphylococcus epidermidis* and *Escherichia coli*, and the residual drug could stably prevent bacterial adhesion. At the same time, it could be expanded to bind other bioactive molecules containing free amino groups such as proteins, combining sustained drug release and long-term antibacterial functions [114].

These studies collectively establish PDA as a cargo-management platform rather than merely a photothermal agent. Polysaccharides alone provide biocompatibility, protein binding, and certain pH- or enzyme-responsive release, but they generally lack the high density of aromatic and quinone groups needed for strong π–π stacking, reversible Schiff-base formation, or Michael addition. PDA, by contrast, offers these functionalities: its aromatic rings enable π–π stacking with aromatic drugs, its quinone groups form pH-sensitive Schiff bases with amines and Michael adducts with thiols, and its catechol groups provide hydrogen bonding and electrostatic interactions [111,112]. Because these interactions differ in strength and pH/redox sensitivity, PDA can decouple the release of multiple cargos according to molecular size and binding affinity [113]. For example, Han et al. demonstrated sequential release of VEGF (fast, Fickian diffusion) and BSA (slow, super Case II transport) from a PDA-containing hydrogel, which would be difficult to achieve with a polysaccharide matrix alone [113]. When combined with polysaccharides, PDA is not merely duplicating matrix functions: the polysaccharide network provides hydration, mechanical support, mucoadhesion, and immunomodulation, while PDA contributes photothermal conversion, high-density binding sites, and stimuli-responsive covalent bonds. This division of labor enables sequential protein delivery, pH-responsive local therapy, and probiotic protection, which are difficult to achieve with AuNRs or polysaccharide matrices alone [76,111,112,113].

Nevertheless, stronger cargo binding may also become a disadvantage if drug desorption is incomplete, and Schiff-base or Michael-addition reactions may alter the activity of amine- or thiol-containing biomolecules [113]. In addition, many reported release studies rely on simplified buffer systems that do not reproduce protein competition, enzymatic degradation, or dynamic pH changes in vivo. Compared with AuNRs, PDA is preferable when sustained release, multidrug loading, and injectable local retention dominate the design requirements. AuNRs remain advantageous when rapid optical triggering and real-time imaging are prioritized. This trade-off should be evaluated using absolute loading content, fraction of therapeutically active cargo released, and in vivo exposure profiles rather than cumulative percentage release alone.

### 5.3. Bone Tissue Engineering and Blood Oxygenation

The PDA systems in this section are not circulating nanocarriers but PDA coatings or interface-engineering materials applied to substrates such as membranes, scaffolds, or sensor surfaces. In bone tissue engineering, Kwack et al. used a film formed by fucoidan and PDA composite as a culture platform for periodontal ligament stem cells (PDLSCs), which could significantly improve the osteogenic potential of cells. RNA sequencing identified 348 differentially expressed genes, and enriched pathways such as actin cytoskeleton regulation were closely related to the osteogenic process [64]. Wu et al. constructed a PDA/β-CD composite coating on the surface of PIM-1 nanoporous membranes through dopamine self-polymerization and β-cyclodextrin hydrogen bonding, significantly improving the hydrophilicity and electronegativity of the membrane, reducing the hemolysis rate to 0.02% and the protein adsorption amount to only 2.7 μg/cm^2^, effectively improving blood compatibility; at the same time, it achieved efficient exchange of O_2_ and CO_2_ with stable performance after 24 weeks of aging [115].

These applications illustrate an additional distinction between PDA and AuNRs: PDA can serve as a durable interfacial modifier even when photothermal treatment is not the principal therapeutic mechanism. Its adhesive coating promotes cell attachment, immobilizes bioactive polysaccharides, and improves membrane hydrophilicity and hemocompatibility. In bone regeneration, the PDA–polysaccharide interface may regulate protein adsorption and cytoskeletal signaling, thereby influencing stem-cell fate [116]. In blood-contacting devices, surface wettability and resistance to nonspecific protein adsorption are more important than heat generation [117].

Such interface-dominated applications are less compatible with AuNRs, whose principal value depends on plasmonic behavior and whose persistent metal content may be undesirable in long-term implants. However, PDA coating thickness and oxidation state must be controlled, because excessive deposition can alter membrane permeability, mechanical properties, and cellular responses [118]. Future work should separate the biological effects of the polysaccharide, PDA chemistry, and substrate topography through appropriate component controls.

### 5.4. Biosensing and Bioadhesion

Chitosan–PDA composites are used in biomedical electrochemical biosensing due to their biocompatibility and adhesion [63]. Costa et al. developed a chitosan–PDA–xanthan gum bio-adhesive with excellent water resistance and adhesion strength, showing potential for wound closure and implant fixation [119].

Biosensing and bioadhesion further demonstrate that PDA’s principal advantage is multifunctional interfacial chemistry. Chitosan contributes film formation and cationic biomolecule binding, PDA provides substrate-independent adhesion and redox-active sites, and xanthan gum can improve rheological stability. These combinations are therefore driven by cooperative interface engineering rather than additive mixing. AuNRs could contribute plasmonic signal enhancement to such systems, but their use would be justified only when optical readout or photothermal actuation provides a clear functional gain.

Overall, polysaccharide-modified PDA systems tend to work best in applications that are chronic, locally administered, or interface-dominated—such as wound healing, sustained drug delivery, tissue engineering, and biomedical functional coatings. Compared to AuNRs, their broad chemical reactivity supports higher cargo loading and more diverse hydrogel architectures, while their biodegradability helps avoid concerns about persistent inorganic residues. On the flip side, these systems suffer from less precise spectral selectivity, variable polymer structure, and potential interference between PDA’s antioxidant properties and ROS-dependent therapies. In short, PDA should not be seen merely as a cheaper alternative to AuNRs; it represents a distinct platform better suited for long-term biological integration rather than rapid plasmonic ablation.

## 6. Comparison and Synergy of Gold Nanorod and Polydopamine Systems

AuNRs are inorganic plasmonic photothermal nanomaterials, while PDA-based platforms can be formulated as systemic nanoparticles, local hydrogels/depots, or coating/interface-engineering materials, depending on the intended therapeutic role. Fundamental disparities exist between the two materials in molecular nature, photothermal generation principles, surface chemistry, in vivo behavior and applicable scenarios. This section conducts a comparative analysis from five dimensions: physicochemical properties, therapeutic performance, pharmacokinetics, biosafety, and hybrid synergistic systems. Instead of merely relying on tables to list parameters, in-depth textual discussions are combined with material mechanisms and practical application performance, while summary tables are supplemented to intuitively present key indicators, achieving complementarity between mechanistic analysis and quantitative data.

### 6.1. Comparison of Physicochemical Properties

Polysaccharide-modified gold nanorods and polysaccharide-modified polydopamine belong to two fundamentally distinct photothermal material systems—inorganic noble metals and organic polymers, respectively. The intrinsic differences in the material substrates directly determine their divergent characteristics in core physicochemical indicators such as photothermal response mechanism, structural stability, modification potential, and biodegradability. These differences also constitute the fundamental logic behind the suitability of the two types of carriers for distinct biomedical scenarios. To intuitively and comprehensively clarify the advantages, disadvantages, and applicability of their physicochemical properties, this section conducts a horizontal comparison across 11 key dimensions, including material nature, photothermal characteristics, structural parameters, modification capability, and biological properties. The specific physicochemical performance parameters are summarized in Table 3.

### 6.2. Therapeutic Properties Comparison

Based on their differentiated physicochemical properties, the two types of polysaccharide-modified nanocarriers exhibit significant scenario-specific differences in core therapeutic functions such as photothermal therapy, drug delivery, antibacterial repair, and immune regulation, with their therapeutic advantages and application limitations being highly complementary. To accurately evaluate their clinical application value and suitable scenarios, this section conducts a comparative overview across eight therapeutic dimensions—including tumor treatment, drug delivery, antibacterial repair, and immune modulation—comprehensively summarizing the therapeutic performance characteristics of the two types of carriers. The specific comparison results are shown in Table 4.

Optical absorption, delivered light, and thermal dose. In comparing the therapeutic performance of AuNRs and PDA, it is important to distinguish three related but different quantities: (i) the intrinsic optical absorption of the nanomaterial, which governs the probability of photon absorption and subsequent heat generation; (ii) the amount of optical energy that actually reaches the target tissue, which depends on tissue composition, scattering, absorption, and irradiation geometry; and (iii) the resulting intratumoral thermal dose, which integrates temperature over time and determines the biological outcome [124,125,126]. AuNRs exhibit wavelength-selective absorption with a tunable longitudinal LSPR, providing high absorption cross-sections at the matching wavelength, but the light delivered at depth is still governed by tissue optics [127]. PDA, by contrast, absorbs broadly across the NIR, offering wavelength flexibility but lower peak absorption efficiency at any given wavelength [128]. Therefore, comparisons based solely on nanomaterial absorption are insufficient; they should be interpreted together with delivered light and thermal dose. These three quantities are further modulated by the pharmacokinetic and biodistribution profiles discussed in the next section, because the amount of nanomaterial that actually reaches the tumor directly influences how much light can be absorbed and converted into heat [129].

### 6.3. Pharmacokinetic and Biodistribution Comparison

Pharmacokinetic (PK) and biodistribution profiles are critical determinants of both therapeutic efficacy and biosafety. However, these properties remain insufficiently characterized for many polysaccharide-modified nanocarrier systems. This section focuses on systemically administered AuNRs and PDA nanoparticles. Local PDA hydrogels, fibers, and coatings follow fundamentally different in vivo kinetics: their performance is governed by local retention time, degradation rate, and tissue integration rather than circulation half-life or EPR-based tumor accumulation. Therefore, PK parameters discussed below should not be extrapolated to local PDA systems.

#### 6.3.1. Circulation Half-Life

Polysaccharide modification significantly prolongs the circulation half-life of nanoparticles compared to their unmodified counterparts, primarily through a stealth effect that reduces uptake by the reticuloendothelial system (RES) [130]. Polysaccharide-based surface coatings, such as dextran and hyaluronic acid (HA), provide steric hindrance and reduce plasma protein adsorption, thereby decreasing opsonization and subsequent RES clearance [61].

For polysaccharide-modified gold nanorods (AuNRs), the circulation half-life ranges from 2 to 12 h, depending on the type of polysaccharide and coating density. Dextran and HA coatings generally confer longer circulation times than chitosan, owing to their neutral or anionic surface charge and superior resistance to protein adsorption. Notably, AuNRs functionalized with both HA and targeting antibodies have demonstrated enhanced tumor targeting efficiencies reaching up to 13% of the injected dose in preclinical models [131].

In the case of polysaccharide-modified polydopamine (PDA) nanoparticles, the circulation half-life ranges from 3 to 18 h. The larger particle size and broader size distribution of PDA may contribute to faster RES clearance relative to monodisperse AuNRs. Nevertheless, PDA is generally regarded as biocompatible, and its reported biodegradability may reduce the risk of chronic tissue retention compared with non-degradable inorganic nanomaterials. However, the degradation rate, metabolic fate, and biological effects of degradation products remain incompletely characterized, and therefore this advantage should be considered tentative rather than unequivocal [132,133].

#### 6.3.2. Biodistribution and Tumor Accumulation

Tumor accumulation for both platforms relies primarily on the enhanced permeability and retention (EPR) effect, with typical accumulation efficiencies ranging from 2% to 10% of the injected dose [134]. Active targeting through polysaccharide–receptor interactions can further enhance tumor accumulation by 2- to 5-fold compared to non-targeted controls [135]. However, the EPR effect is highly variable—it is markedly more pronounced in small animal models than in human tumors, where tumor heterogeneity, variable vascular density, and high interstitial fluid pressure significantly reduce EPR-mediated accumulation [136]. Active targeting through polysaccharide–receptor interactions can further enhance tumor accumulation by 2- to 5-fold compared to non-targeted controls, but this effect depends strongly on receptor expression levels, ligand density, polysaccharide molecular weight, stability of surface presentation, and potential interference from protein corona formation; receptor expression alone is not sufficient to guarantee improved delivery [135].

Regarding organ distribution, both types of nanoparticles predominantly accumulate in the liver and spleen, i.e., the RES organs [137]. AuNRs exhibit higher and more persistent liver accumulation due to their non-degradable gold core, whereas PDA is believed to be more readily metabolized and cleared from these organs, although its degradation kinetics and metabolic fate remain incompletely defined [138,139]. Notably, the slow elimination kinetics of gold nanoparticles can lead to long-term accumulation in the liver and spleen, with inflammation and fibrotic responses reported in vital organs following chronic exposure [121].

With respect to clearance pathways, polysaccharide coatings are degraded by endogenous enzymes [140]. PDA is believed to undergo degradation via enzymatic oxidation and lysosomal pathways, although the exact metabolic mechanisms remain incompletely characterized [138]. In contrast, gold from AuNRs is primarily cleared through the hepatobiliary pathway, with very slow excretion kinetics that can extend over weeks to months [141,142].

### 6.4. Comparison of Biosafety Performance

Beyond pharmacokinetics and biodistribution, biosafety encompasses the biological impact of nanocarriers at cellular, tissue, and systemic levels. This section compares the two platforms in terms of in vitro cytotoxicity, immunogenicity, hematological compatibility, and organ-level toxicity.

#### 6.4.1. Biosafety of Polysaccharide-Modified Gold Nanorods

The primary source of AuNR cytotoxicity is residual CTAB, a cationic surfactant that disrupts cell membranes [143]. Replacing CTAB with biocompatible coatings—PEG, hyaluronic acid, phospholipids, or 11-mercaptoundecanoic acid—significantly reduces in vitro toxicity while maintaining colloidal stability [144]. However, the anisotropic rod shape itself may induce higher cytotoxicity than spherical counterparts due to enhanced membrane penetration.

Immunogenicity is a critical concern: a 2024 study demonstrated that PEGylated AuNRs specifically activate the NLRP3 inflammasome in differentiated THP-1 macrophages, a shape-dependent response not seen with nanospheres or nanostars [145]. This activation downregulates sterol/cholesterol biosynthesis and oxidative phosphorylation while upregulating PON2, a key oxidative stress regulator. In genotoxicity assays, AuNRs induce indirect oxidative DNA damage in mammalian cells rather than direct DNA interaction [146].

At the organ level, chronic exposure studies in mice demonstrate that gold nanorods undergo prolonged retention in the liver and spleen with progressive pathological changes. Fernandez Alarcon et al. showed that gold nanorods persisted in liver sinusoidal endothelial cells and Kupffer cells for up to 47 days without degradation [147]. Jakic et al. reported BSA-coated gold nanoparticles retained in the liver, spleen, and kidneys for 120 days, with renal inflammation and fibrosis despite minimal gold deposition [121]. Soltysova et al. demonstrated that 10 nm PEGylated gold nanoparticles were still detectable in mice 120 days after a single intravenous injection, primarily in the liver, spleen, and kidneys [148]. The non-degradable gold core raises concerns about chronic oxidative stress, as studies have demonstrated that gold nanorods can induce oxidative stress and genotoxicity in mammalian cells, with effects dependent on particle size, shape, and surface chemistry [149]. However, Pincela Lins et al. reported that appropriately coated gold nanorods—functionalized with PEG, citrate, or cell membranes—do not necessarily elevate intracellular ROS levels, and the primary toxicity source remains residual CTAB surfactant, which is inherently cytotoxic [69]. Concerns regarding potential blood–brain barrier crossing by sub-10 nm particles remain incompletely validated.

#### 6.4.2. Biosafety of Polysaccharide-Modified Polydopamine Nanoparticles

Polysaccharide-modified polydopamine (PDA) nanoparticles are generally regarded as favourable biocompatibility, with most studies confirming no toxicity even at high concentrations, and their intrinsic radical-scavenging activity further attenuates oxidative stress [123,150]. However, residual unreacted dopamine monomers can elicit acute cytotoxicity if not thoroughly removed. A size-dependent study revealed that approximately 100 nm PDA nanoparticles exhibited the lowest cytotoxicity, whereas smaller (approximately 50 nm) and larger (approximately 200 nm) particles provoked stronger toxic effects [70]. The most definitive long-term in vivo evidence comes from Szukalska et al., who observed dose-dependent oxidative stress in mouse liver and kidneys following mesoporous PDA treatment. PDA has been reported to be biodegradable via enzymatic oxidation into melanin-like products, which may reduce the risk of persistent organ accumulation [95]. However, its degradation rate, metabolic fate, and the immunogenicity of degradation products remain poorly characterized, and therefore biodegradability should be regarded as a potential rather than an established safety advantage. The protein corona formed on PDA nanoparticles can obscure targeting ligands and alter biodistribution [151]. Future studies should adopt standardized absorption, distribution, metabolism, and excretion (ADME) profiling and ≥6-month chronic toxicity tests to support clinical translation.

### 6.5. Synergistic Strategies

AuNR–PDA hybrids integrate the tunable plasmonic properties of AuNRs with the broadband absorption and adhesive chemistry of PDA. However, hybridization is only justified when it provides a demonstrable advantage over either component alone; otherwise, the added structural complexity may introduce new variables in synthesis, characterization, and safety assessment without corresponding therapeutic benefit. This section evaluates the evidence for such advantages.

Optical behavior. PDA coating significantly alters the plasmonic properties of AuNRs. It decreases the longitudinal plasmon peak while increasing the transversal peak, and the LSPR peak can be tuned from ~750 to ~950 nm by adjusting the PDA shell thickness [152]. This tunability enables optimization for specific NIR windows but also means that the optical response of the hybrid cannot be predicted from the AuNR core alone.

Shell thickness and heat transfer. In AuNR–PDA hybrid systems, photoexcitation of the AuNR core generates hot carriers via localized surface plasmon resonance (LSPR) damping. The PDA shell can accept these hot carriers, facilitating charge transfer from the AuNR to PDA while suppressing carrier recombination and shuttling them to the particle surface for reactive oxygen species (ROS) generation [153]. This interfacial charge/energy transfer, combined with the low thermal conductivity of PDA that provides slow thermalization of the plasmonic heat, contributes to an enhanced overall photothermal effect [153]. Shell thickness is a critical parameter for AuNR–PDA hybrids. An intermediate shell thickness is generally considered optimal for balancing hot-carrier transfer and thermal insulation, whereas excessive PDA deposition may cause nanoparticle instability and reduced photothermal conversion efficiency [153]. Optimal thickness balances hot-carrier transfer and thermal insulation, while excessive PDA deposition may compromise nanoparticle stability and reduce photothermal conversion efficiency. These synergistic effects underpin the enhanced photocatalytic and photothermal performance of AuNR–PDA hybrids relative to either component alone [153].

Cargo loading and pharmacokinetics. PDA hybridization substantially improves drug loading and prolongs circulation compared with bare AuNRs. These advantages arise from π–π stacking, mesoporous structures, and reduced immune clearance. Qi et al. achieved 86.16% doxorubicin loading and pH/NIR dual-triggered release using a PDA–AuNR/graphene oxide hybrid, demonstrating that the PDA layer provides loading capacity that bare AuNRs cannot match [153]. Wang et al. also reported a PDA-coated AuNR system with dual pH/NIR-responsive doxorubicin release for high-efficiency cancer treatment [154].

Therapeutic performance and imaging. Side-by-side comparisons show that the hybrid outperforms both small and large AuNRs in cell ablation and structural stability [155]. Aguilar-Ferrer et al. prepared core–shell PDA-coated AuNRs with enhanced photothermal and photocatalytic activity [153]. PDA coating also protects against laser-induced melting, preserving photoacoustic signals. Yim et al. demonstrated that PDA coating significantly enhanced photoacoustic signal intensity and photothermal efficiency compared with bare AuNRs, with the hybrid achieving 95% ablation of SKOV3 ovarian cancer cells versus 66% for large AuNRs and 74% for small AuNRs [155].

Multifunctional integration. The hybrid enables functions neither component achieves alone, including gene/photothermal combination therapy, SERS imaging, and pH/NIR dual-triggered release. Niu et al. designed PDA/gold nanorod-based nanoparticles for synergistic genetic and photothermal combination therapy [156]. Zhang et al. used PDA-coated AuNRs in liquid crystal elastomer actuators for fast NIR-driven movement, illustrating the broader functional versatility of the hybrid [157].

When is hybridization advantageous? Hybridization is beneficial when: (i) shell thickness is optimized (approximately 10–15 nm); (ii) high drug loading is required; (iii) prolonged circulation is needed; (iv) structural stabilization against melting is critical; and (v) multimodal imaging or gene/drug co-delivery is desired. Conversely, when spectrally selective heating at a precise LSPR wavelength is paramount, the bare AuNR may be preferable because the PDA shell can damp the plasmonic peak and reduce spectral selectivity.

Finally, Table 5 provides a concise study-level summary of representative polysaccharide-modified AuNR and PDA systems, categorized by PDA architecture (systemic nanoparticles, local hydrogels/depots, and coatings/interface materials). These examples illustrate the practical implementation of the design principles and comparative trends discussed in this section, and complement the quantitative comparison in Table 3 and Table 4.

## 7. Challenges and Future Directions

### 7.1. Current Core Challenges

Although Section 2 proposes a function-oriented framework for polysaccharide selection, its predictive validity has not yet been established through rigorous head-to-head comparisons. Most studies evaluate only one polysaccharide–carrier combination, making it difficult to separate the effects of polysaccharide identity, molecular weight, substitution degree, grafting density, and carrier architecture. Future studies should compare multiple polysaccharides under matched particle size, surface coverage, drug dose, and irradiation conditions. Receptor targeting, administration-route compatibility, microenvironment responsiveness, and protein-corona stability should be treated as experimentally testable design variables rather than assumed properties [158,159,160]. Such comparative datasets are essential for converting the proposed framework from a qualitative guide into a predictive selection model. For example, HA and chondroitin sulfate should be prioritized for CD44-associated targeting only after receptor expression and ligand accessibility have been verified; dextran may be preferable for intravenous stealth, whereas chitosan is more suitable for mucosal delivery [58,161]. Alginate and other gel-forming polysaccharides are better suited to local depots, while heparin requires careful control of anticoagulant activity when used for growth-factor delivery [162].

Biosafety and metabolism. Polysaccharide modification improves biocompatibility, but risks persist [163]. Ultrasmall gold nanoparticles may cross biological barriers, but whether conventional polysaccharide-modified AuNRs exhibit clinically relevant blood–brain barrier penetration remains insufficiently established [164]. For PDA, unreacted dopamine monomers are cytotoxic, and the metabolic fate of degradation products remains poorly characterized [70,165]. Residual coupling reagents and reaction by-products may cause cytotoxicity or inflammatory responses if purification is inadequate. Balancing modification stability with minimal reagent residue remains unresolved.

NIR-II wavelengths generally exhibit reduced scattering and can improve penetration relative to NIR-I, although the clinically achievable treatment depth remains strongly dependent on tissue composition, irradiation geometry, and the required thermal dose. Temperatures exceeding 50 °C are required for tumor ablation, yet uncontrolled thermal diffusion causes collateral burns and coagulative necrosis. Irradiation parameters vary widely across studies—power density 0.5–2.0 W/cm^2^, duration 2–20 min, continuous versus pulsed mode, ΔT 10–40 °C—preventing meta-analysis. Neither a minimum effective photothermal dose for tumor ablation nor a standardized safety guideline exists.

To better define these dosimetric issues, the laser power densities typically required for the discussed materials should be specified. For photothermal gold nanorods (AuNRs), reported values span 0.75–2.3 W/cm^2^ depending on surface chemistry and geometry: Au@PEG nanorods achieve 53.3 °C at 793 nm and 1 W/cm^2^ within 9 min; AuNRs@BSA reach 85.5 °C at 1064 nm and 0.75 W/cm^2^; and AuNRs-Alb-NPs rise from 26 to 61 °C at 808 nm and 1.5 W/cm^2^ within 5 min [166,167]. In vivo, an 808 nm laser at 1.6 W/cm^2^ for 15 min causes irreversible thermal damage to prostatic tumours, and 2.3 W/cm^2^ for 15 min is similarly effective [166]. For polysaccharide-modified polydopamine (PDA) systems, reported power densities range from 0.48 to 3.0 W/cm^2^: PDA nanoparticles reach 53 °C at 808 nm and 0.48 W/cm^2^ within 300 s, and ultrasmall PEGylated PDA nanoparticles at 3 W/cm^2^ for 10 min effectively destroy 4T1 cells [168]. These values are 10–100-fold higher than those commonly used in clinical PDT (4–150 mW/cm^2^), because PTT requires macroscopic heating (ΔT ≥ 10–20 °C) whereas PDT depends on photochemical ROS generation [169]. The ANSI Z136.1 maximum permissible skin exposure for 600–1000 nm is 200–800 mW/cm^2^, which most PTT conditions exceed [170]. Without nanomaterials, normal tissue tolerance to NIR light is limited by intrinsic absorption—skin is a stronger NIR absorber than most tissues owing to melanin and haemoglobin—and for a 976 nm diode laser used in soft-tissue surgery, thermal damage was reported at 208.40 ± 133.81 µm in the epithelium and 330.14 ± 147.45 µm in the connective tissue (4–6 W, CW) [171]. Standardized reporting of wavelength, power density, irradiation time, beam size, and mode, together with in situ thermometry, is therefore needed.

Treatment standardization. Although approximate temperature ranges are often cited—such as ~42–45 °C for apoptosis and >50 °C for coagulative necrosis—these values should be regarded as rough, context-dependent guidelines rather than universal thresholds. No consensus defines the precise temperature threshold for ICD versus necrosis, or the hyperthermia duration required for synergistic chemotherapy. Reported optimal temperatures for ICD vary widely depending on cell type, heating rate, and thermal dose. In a murine neuroblastoma model, ICD markers were maximally expressed at 63.3–66.4 °C, whereas both lower (50.7–52.7 °C) and higher (83.0–83.5 °C) temperatures yielded markedly lower ICD induction [172]. In a triple-negative breast cancer model, temperatures below 45 °C were insufficient to induce significant apoptosis, whereas an optimal range of 50–60 °C was identified for balancing cell killing and ICD [173]. Conversely, silica-coated gold nanorods induced potent ICD at mild temperatures of 44–45 °C in colon tumor models [174]. Regarding thermal dose, the clinically established metric CEM43 has thresholds for complete thermal necrosis ranging from 25 to 240 CEM43 depending on tissue type [79]. Future studies should therefore report thermal dose parameters (e.g., CEM43) together with temperature–time profiles, rather than relying on fixed temperature cut-offs. Furthermore, reference materials for standardizing photothermal conversion efficiency measurements—such as gold nanorods with well-characterized optical properties—remain lacking, and standardized in vivo protocols for evaluating photothermal efficacy are absent [175].

Manufacturing and stability. Seed-mediated AuNR synthesis yields batch-to-batch coefficient of variation > 15% [176]. PDA polymerisation is difficult to control, producing inconsistent particle size [177]. Thiolated polysaccharide conjugation efficiency on AuNRs typically reaches only 40–60%. Drug loading for chemotherapeutics in AuNR systems often falls below 5%. Intravenous AuNRs may cause thrombosis; PDA hydrogels require precise tuning of mechanical strength and degradation rate.

Regulatory obstacles. Long-term toxicity, immunogenicity, and metabolic data are insufficient for regulatory evaluation. While general regulatory frameworks for nanomaterials exist—including the U.S. Food and Drug Administration (FDA) guidance “Drug Products, Including Biological Products, that Contain Nanomaterials” and the European Medicines Agency (EMA)’s reflection papers on nanotechnology-based medicinal products—no platform-specific guidance has been issued for polysaccharide-modified AuNR or PDA systems. Notably, gold nanorods are not yet in human clinical trials, although Sona Nanotech has completed an FDA-required preclinical toxicity study, a prerequisite for initiating clinical trials. To date, no polysaccharide-modified AuNR or PDA formulation has received marketing approval from the FDA or EMA.

### 7.2. Future Perspectives

#### 7.2.1. Material Design and Functional Precision

Future advances demand multidimensional modifications to achieve precise functional regulation. In AuNR systems, polysaccharide–metal ion composite modifications—such as alginate–Ca^2+^—can be employed to construct photothermal–ion synergistic therapy platforms, where Ca^2+^ release promotes tumor cell apoptosis while simultaneously enhancing colloidal stability [178]. In PDA systems, responsive crosslinking motifs—for instance, ROS-sensitive disulfide bonds—can be incorporated to enable controlled degradation and drug release of polysaccharide–PDA composite carriers specifically within the tumor microenvironment [179]. Furthermore, the development of novel polysaccharide derivatives (e.g., sulfated dextran, chitosan grafted with peptide segments) may improve targeting specificity and circumvent receptor-mediated competitive inhibition [180].

Multifunctional integration represents a key developmental trajectory. A representative configuration is the photothermal–photodynamic–immune triple synergistic system: AuNRs serve as the photothermal core, PDA acts as a carrier for photosensitizers such as porphyrin to enable photodynamic therapy, and polysaccharides such as Astragalus polysaccharide function as immunomodulators to activate dendritic cells [181,182]. Upon near-infrared (NIR) irradiation, all three therapeutic mechanisms are triggered simultaneously, achieving not only tumor ablation but also inhibition of recurrence and metastasis [49]. Concurrently, the incorporation of intelligent response elements—such as pH/enzyme dual-responsive polysaccharides—enables spatiotemporally precise regulation of drug release, thereby further reducing off-target toxicity [183].

#### 7.2.2. Preparation Process and Formulation Optimization

Continuous-flow technologies have been established for some pharmaceutical and nanoparticle manufacturing processes and may offer a route to improve reproducibility; however, their suitability for polysaccharide-modified AuNR/PDA systems remains to be demonstrated [184,185,186]. Template-based methods improve PDA polymerisation control: DNA-origami-templated polymerisation achieves nanometre-resolution control over PDA shape and size, and template-assisted approaches can preserve the designed morphology of dopamine-modified polymers [187,188]. Green modification processes such as enzyme-catalysed crosslinking warrant further investigation, building on the established role of horseradish peroxidase (HRP) in green chemistry—HRP-catalysed crosslinking has been applied to protein-based hydrogels and even to phenolic-polysaccharide derivatives on cell surfaces [189,190]. Formulation innovations include injectable microspheres for locoregional therapy, exemplified by biodegradable microspheres delivering sustained locoregional chemotherapy in transarterial chemoembolisation and injectable tumour-responsive hydrogels for local chemotherapy; wearable photoresponsive dressings for chronic wounds, supported by photothermal antibiofilm hydrogels effective against infected diabetic wounds; and multilayer polysaccharide-coated nanocapsules for oral delivery, as demonstrated by layer-by-layer chitosan-coated nanoliposomes and polysaccharide-coated nanoparticles that protect oral insulin against the gastric environment [191,192,193]. Lyophilised formulations stable at room temperature would reduce cold-chain dependency and enhance clinical practicality; freeze-drying has already enabled stable storage of nanocarrier and viral-vector formulations outside the deep-freeze cold chain [194].

#### 7.2.3. Clinical Translation and Interdisciplinary Integration

Targeted preclinical investigations should prioritise disease models that recapitulate clinical conditions. Patient-derived xenograft (PDX) models more faithfully preserve tumour histology, heterogeneity and microenvironment than conventional cell-line models and are increasingly used to evaluate nanomedicines [194,195]. Diabetic infected-wound models, widely employed to assess photothermal antimicrobial dressings, likewise provide clinically relevant endpoints. Rigorous evaluation of long-term carrier safety and efficacy is imperative, as current nanotoxicological understanding is largely based on short-term studies, while metabolomics offers a complementary high-throughput route to map systemic perturbations [196]. Treatment monitoring should integrate imaging with biomarker panels, consistent with theranostic strategies that combine imaging and therapy in a single nanoplatform [197]. Parallel efforts should standardise nanocarrier characterisation—dynamic light scattering (DLS) for particle size, surface plasmon resonance for optical behaviour, and X-ray photoelectron spectroscopy for surface modification degree [198].

Interdisciplinary collaboration will continue to catalyze innovation. Artificial intelligence and machine learning can be leveraged to optimize carrier design and predict how polysaccharide modifications influence AuNR and PDA performance. Three-dimensional bioprinting enables the fabrication of polysaccharide–PDA tissue engineering scaffolds that precisely match tissue defect geometries [199]. Integration with wearable devices allows personalised regulation of NIR dosage; temperature-feedback photothermal systems already enable real-time, personalised control of light dose [200]. Beyond oncology, application fields should be expanded: in atherosclerosis, photothermal gold-nanorod platforms enable plaque-targeted ablation combined with anti-inflammatory therapy; in nerve repair, polydopamine-modified nerve guidance conduits incorporating electrical cues promote axonal regeneration [201,202].

#### 7.2.4. Key Strategies to Address Challenges

The translational strategies discussed below represent our proposed framework based on current preclinical evidence and are not intended to be formal regulatory requirements. Specific regulatory expectations should be confirmed with the appropriate agencies.

We suggest that the clinical translation of polysaccharide-functionalized AuNR and PDA nanocarriers would benefit from moving beyond empirical formulation adjustments toward standardized, metrology-driven translational frameworks. Inconsistencies in photothermal efficacy reported across preclinical studies stem largely from reliance on nominal incident laser power and exposure durations, which fail to capture tissue-specific optical attenuation, scattering variations, and dynamic spatial heat dissipation. We recommend implementing standardized thermal dosimetry metrics—primarily cumulative equivalent minutes at 43 °C—as a practical approach that may provide a useful baseline for cross-study validation and clinical dose scaling [175]. We further propose that integrating near-infrared therapeutic lasers with non-invasive, real-time spatial thermometry (such as photoacoustic thermometry or magnetic resonance thermometry) could establish closed-loop feedback systems. This integration may enable automated power modulation to maintain target tissue heating within therapeutic margins—such as 41–43 °C for vascular permeabilization and radiosensitization, or 43–47 °C for immunogenic ablation—while helping to prevent thermal damage to adjacent margins [203].

From a manufacturing standpoint, conventional flask-based batch synthesis of anisotropic gold nanorods and oxidative PDA self-assembly is often challenged by batch-to-batch variations in aspect ratio, shell thickness, and surface defect densities. We suggest that transitioning to droplet-based microfluidics and continuous-flow reactors may provide improved control over reagent diffusion, local concentration gradients, and thermal uniformity [204]. In our view, implementing in-line process analytical technologies (PAT)—including real-time extinction spectroscopy and multi-angle dynamic light scattering—could enable continuous monitoring of critical quality attributes during production. This continuous verification may help ensure tight tolerances on longitudinal surface plasmon resonance peak positions, hydrodynamic dimensions, polysaccharide grafting densities, and the complete removal of toxic residual surfactants (such as reducing free CTAB to sub-ppm levels verified by mass spectrometry) [205].

Long-term tissue persistence and unclear biological clearance pathways remain major regulatory hurdles for non-biodegradable gold cores and structurally complex PDA networks. We propose that future development may prioritize “clearance-by-design” architectures that actively avoid irreversible reticuloendothelial system bioaccumulation [205]. This can be accomplished through modular core–satellite nanoconstructs, wherein sub-5-nm gold nanocrystals or ultrasmall PDA dots are assembled onto biodegradable polysaccharide backbones via microenvironment-cleavable bonds (such as glutathione-cleavable disulfides or enzyme-sensitive peptide linkers) [206]. Upon reaching the target tissue and encountering intracellular catabolic cues, these assemblies break down into individual components below the ~5.5 nm glomerular filtration threshold, enabling renal excretion and minimizing long-term parenchymal retention [207].

We suggest that toxicological safety testing should also shift beyond basic 24-h cell viability assays, which overlook subtle cellular stress, immunogenicity, and chronic organ accumulation. Rigorous characterization of the biomolecular corona that forms around polysaccharide-modified surfaces in human biofluids allows researchers to optimize polymer charge and branching to enrich dysopsonins while suppressing complement activation cascades [208]. In parallel, we recommend that long-term safety profiles be established using longitudinal multi-omics, single-cell RNA sequencing, and spatial transcriptomics in clearance organs (liver, spleen, and kidneys) over extended post-treatment windows to confirm the absence of latent fibrosis, immune perturbation, or chronic metal toxicity [209].

As a general framework, we propose a stepwise translational pathway that may be adapted according to the specific product and intended indication. In the short term, formulation optimization and analytical characterization should be completed, followed by pharmacokinetic and toxicological evaluation in rodents (e.g., approximately 6-month repeated-dose studies) to identify dose-limiting toxicities and major organ accumulation. In the medium term, longer-term safety and efficacy studies in large animals (e.g., ≥12 months) may be considered to assess chronic exposure, immune responses, and clearance profiles. In parallel, GMP-compatible scale-up should be addressed, with emphasis on batch-to-batch reproducibility, removal of residual surfactants, and in-process quality control. In the long term, if these stages are successful, a first-in-human study could be designed for a selected indication with a favorable optical access and clear clinical need. This multi-year pathway is proposed as a general framework and does not represent formal regulatory requirements; specific study designs, durations, and milestones should be confirmed with the appropriate regulatory agencies.

Within this framework, initial clinical translation should focus on disease indications with clear optical access, mitigating the physical limitations of deep-tissue light penetration. Applications such as superficial bladder carcinomas, head and neck malignancies, intraoperative tumor margin sterilization, and recalcitrant localized biofilms present manageable optical paths for precise laser delivery. Demonstrating reproducible efficacy and manageable safety profiles in these accessible settings may help generate the standardized preclinical data that are likely to support regulatory filings and phase I clinical trials, although specific requirements should be confirmed with the relevant agencies.

## 8. Conclusions

Polysaccharide-modified AuNR and PDA-based systems represent two complementary strategies for constructing NIR-responsive biomedical systems. Polysaccharide modification can improve colloidal stability, reduce nonspecific biological interactions, introduce receptor-mediated targeting, and enable microenvironment-responsive delivery. However, these benefits depend strongly on polysaccharide identity, molecular weight, charge density, grafting method, and surface presentation.

AuNR-based platforms provide spectrally tunable and rapid photothermal conversion together with strong photoacoustic, scattering, and CT imaging potential. They are therefore particularly suitable for image-guided tumor ablation, localized antibiofilm treatment, and applications requiring precise optical triggering. In contrast, PDA offers broad chemical functionality, high cargo-loading capacity, tissue adhesion, antioxidant activity, and compatibility with injectable hydrogels and regenerative matrices. PDA-based systems are thus better suited to sustained local delivery, wound healing, tissue engineering, and other interface-dominated applications. Hybrid AuNR–PDA architectures may combine these advantages, but their added structural complexity must provide a demonstrable therapeutic benefit.

Platform selection should therefore be guided by the therapeutic objective, administration route, required optical precision, cargo-loading and release profile, disease microenvironment, and long-term safety rather than by photothermal efficiency alone. Future translation will require standardized characterization, realistic disease models, robust pharmacokinetic and chronic-toxicity evaluation, and reproducible GMP (Good Manufacturing Practice)-compatible manufacturing. A function-oriented design framework may help advance polysaccharide-modified AuNR and PDA systems from proof-of-concept formulations toward more predictable biomedical technologies.

## Figures and Tables

**Table 1:** Key physicochemical features, potential biological interactions, stimulus responsiveness, and representative applications.

Polysaccharide	Key Features	Stimulus Responsiveness	Typical Applications in AuNR/PDA Systems
Hyaluronic acid (HA)	Anionic; –COOH, –OH; CD44 targeting	pH/enzymatic	Tumor targeting, wound healing, antibacterial [25,26]
Chitosan (CTS)	Cationic; –NH_2_; mucoadhesion, gene binding	pH (protonation)	Gene delivery, photothermal ablation, antibacterial [54]
Dextran	Neutral; –OH; stealth/long circulation	Enzymatic	Immunomodulation, antioxidant, prodrug carriers [55]
Alginate	Anionic; –COOH; –	pH (protonation)	Nanogels, ionic crosslinking, core stabilization [38]
Pullulan	Neutral; –OH; hepatic targeting, film-forming	Chemically modifiable	Film-forming matrices, hydrogels, local photothermal dressings [56]
Chondroitin sulfate	Anionic; –SO_3_H, –COOH; CD44 targeting, cartilage repair	pH/enzymatic	Joint repair, tissue engineering, anti-inflammation [57,58]
Heparin	Strongly anionic; –SO_3_H, –COO^−^; VEGF/FGF binding	Non-responsive (primarily ligand-binding)	Angiogenesis, soft tissue regeneration, TME modulation [59,60]
*Poria cocos* polysaccharide	Neutral; β-glucan; immunomodulatory	Enzymatic	Photothermal immunotherapy, immune activation [17]
*Bletilla striata* polysaccharide	Neutral; glucomannan; biocompatible, hemostatic	Enzymatic	Wound healing, diabetic wound repair, CO_2_ release [18]
*Belamcanda chinensis* polysaccharide	Anionic; Man/Glc/Gal; antitumor, antioxidant	Enzymatic	Photothermal cancer therapy [47]
Astragalus polysaccharide	Anionic; immunostimulatory	Enzymatic	Photothermal immunotherapy, DC maturation, T-cell activation [50]
Glycyrrhiza polysaccharide	Anionic; immunomodulatory	Enzymatic	Vaccine adjuvant, DC vaccine, antitumor immunity [51]
Porphyran	Anionic; sulfated galactan; antioxidant	pH/enzymatic	pH-responsive photothermal immunotherapy, immune activation [52]
*Pleurotus ferulae* polysaccharide	Neutral; β-glucan; immunomodulatory	Enzymatic	DC maturation, antitumor immunity, immunomodulation [53]

**Table 2:** A function-oriented decision pathway for selecting polysaccharide-modified AuNR/PDA systems. (This table is intended as a practical guide. Final selection should also consider molecular weight, charge density, degree of substitution, degradability, and batch reproducibility. AuNR and PDA data are not directly comparable across all categories; comparisons should be made within the same administration route and formulation type.).

Decision Step	Key Variables/Options	Polysaccharide Role	AuNR vs. PDA Relevance
Administration route	Systemic, local, topical	Affects stealth, circulation time, and targeting requirements [16,25,61]	AuNRs often used for systemic tumor targeting; PDA frequently used for local depots or coatings [18,62,63,64]
Biological barrier	Tumor extracellular matrix (ECM); mucus; biofilm; blood–brain barrier (BBB)	Determines need for mucoadhesion, targeting, or penetration [16,65,66]	Chitosan–AuNR for biofilm; HA–AuNR for CD44+ tumors; PDA coatings for mucosal surfaces [10,25,67]
Required function	Targeting; stealth; mucoadhesion; stimulus responsiveness; imaging; sustained release	Provides targeting ligands (HA), stealth (dextran), mucoadhesion (chitosan) [24,27,36]	AuNRs add tunable LSPR and imaging; PDA adds adhesive/coating and broad absorption [7,8,9,10]
Polysaccharide choice	HA; chitosan; alginate; dextran; molecular weight (MW); charge density	Controls colloidal stability, targeting, immune interactions [16,61,65]	Polysaccharide on AuNR affects targeting and toxicity; on PDA, it governs surface properties [10,25,68]
Carrier architecture	AuNR core; PDA nanoparticle; PDA hydrogel/coating/scaffold	Polysaccharide shell determines surface properties on AuNRs; in PDA hydrogels/coatings, the polysaccharide matrix provides mechanical support and hydration [17,18,62,63]	AuNRs are discrete nanoparticles; PDA can be nanoparticle or interfacial coating [7,8,9,63,64]
NIR requirement	NIR-I (700–950 nm) vs. NIR-II (1000–1700 nm); power density; exposure time; thermal dose	Minimal direct influence; mainly affects colloidal stability and surface presentation	AuNRs have narrow tunable absorption; PDA absorbs broadly but is less efficient [4,6,7,10]
Main safety constraint	Long-term gold retention; uncertain PDA degradation fate; potential immunogenicity	Polysaccharide can reduce toxicity and immunogenicity [16,61,69]	AuNRs may pose long-term retention concerns; PDA degradation rate uncertain [70,71]

**Table 3:** Comparison of key performance metrics of polysaccharide-modified AuNR and PDA nanocarriers. (Values are compiled from representative original studies and are intended for qualitative comparison only. They depend strongly on particle size, surface chemistry, formulation, administration route, animal model, irradiation conditions, and experimental design. Unless otherwise stated, the data are from polysaccharide-modified AuNR/PDA systems; where broader nanomaterial literature is cited, this is indicated. Direct quantitative comparison between studies should be made with caution.).

Property	Polysaccharide-Modified AuNRs	Polysaccharide-Modified PDA
Material class	Inorganic (noble metal) [1]	Organic (polymeric) [9,10]
Photothermal mechanism	Localized surface plasmon resonance (LSPR) [7]	Broadband absorption, non-radiative relaxation
Absorption profile	Sharp LSPR peak, tunable by aspect ratio	Broad absorption across UV-Vis-NIR
Photothermal conversion efficiency	20–30% at LSPR peak (Poria cocos–AuNRs; Belamcanda chinensis–AuNRs; thiolated lemon polysaccharide–AuNRs) [17,47,82]	5–30%, wavelength-dependent (hydroxyethyl starch-stabilized PDA; chitosan oligosaccharide/oxidized herb polysaccharide/mesoporous PDA hydrogel) [12,111]
Photostability	Moderate; polysaccharide-modified AuNRs show some risk of shape deformation at high power [17,47,82]	Excellent; pectin/PDA hydrogels show no significant photobleaching, degradation, or loss of functionality over five successive NIR on/off cycles [75]
Typical particle size	Typically tens of nanometres in length, with diameter and aspect ratio dependent on synthesis (polysaccharide-modified AuNRs) [17,47]	50–500 nm (nanoparticles); bulk hydrogels (Bletilla striata polysaccharide–PDA; chitosan/alginate–PDA) [18,120]
Surface functionalization	Au–S bonds, electrostatic adsorption, amide bonds [17,65,72]	Michael addition, Schiff base, hydrogen bonding, π–π stacking [10,68,75,76]
Functional group density	Limited by surface area and conjugation chemistry	High; abundant catechol, amine, quinone groups [9,10]
Biodegradability	Non-degradable gold core; polysaccharide shell degradable [11,121]	Potentially biodegradable; degradation rate and long-term metabolic fate remain incompletely defined [12]
Imaging capability	Excellent (PA imaging, dark-field microscopy, CT) [8]	Moderate (PA imaging) [122]
Drug loading capacity	Low–moderate for surface adsorption (<5%); can be enhanced by mesoporous or multilayer coatings (HA/hydroxyethyl chitosan–AuNRs) [86]	Moderate to high (30–40%), particularly for mesoporous structures (polysaccharide–PDA hydrogel) [111]

**Table 4:** Comparison of therapeutic performance between polysaccharide-modified gold nanorods and polysaccharide-modified polydopamine. (Values in this table are compiled from representative original studies and are intended for qualitative comparison only. Therapeutic performance depends strongly on particle size, surface chemistry, formulation, administration route, animal model, irradiation conditions, and experimental design. Unless otherwise stated, the data refer to polysaccharide-modified AuNR/PDA systems; where broader nanomaterial literature is cited, this is indicated. Where comparable quantitative evidence is limited, qualitative comparisons are used. Direct quantitative comparison between studies should be made with caution.).

Therapeutic Aspect	Polysaccharide-Modified AuNRs	Polysaccharide-Modified PDA
PTT efficacy	High; rapid, efficient heating at LSPR wavelength (Poria cocos–AuNRs; Belamcanda chinensis–AuNRs; thiolated lemon polysaccharide–AuNRs) [17,47,82]	Moderate–high; broadband but lower peak efficiency (hydroxyethyl starch-PDA; polysaccharide–PDA hydrogel) [12,111]
PDT capability	Limited; requires additional photosensitizer (porphyran-coated AuNRs; Poria cocos–AuNRs) [17,52]	Dependent on photodynamic/ROS activity; often requires a photosensitizer, whereas PDA may also scavenge ROS depending on oxidation state and polysaccharide matrix [123]
Tumor accumulation	Moderate; dependent on EPR effect and active targeting (HA-modified AuNRs) [25]	Moderate; similar EPR dependence (polysaccharide–PDA hydrogel) [111]
Tumor penetration	Limited by particle size; polysaccharide modification (e.g., chitosan, HA) can improve penetration via receptor-mediated transport (HA/hydroxyethyl chitosan–AuNRs; Janus chitosan–gold nanoparticles provide supporting evidence for improved cellular uptake) [84,86]	Variable; PDA nanoparticles similar to AuNRs; hydrogels for local delivery (chitosan/alginate–PDA) [120]
Sustained release	Limited; surface-adsorbed drugs release rapidly (HA/hydroxyethyl chitosan–AuNRs) [86]	Excellent; mesoporous structure enables sustained release (dextran–PDA hydrogel) [107]
Antibacterial efficacy	High photothermal killing; limited intrinsic activity (fucose-containing polymer–AuNRs) [67]	High photothermal killing + intrinsic PDA antibacterial activity (chitosan–PDA; curdlan–PDA) [104,114]
Wound healing	Moderate; primarily photothermal antibacterial (polysaccharide–AuNR hydrogel) [102]	Excellent; photothermal + antioxidant + biomimetic matrix (Bletilla striata polysaccharide–PDA; dextran–PDA hydrogel) [18,107]
Immunomodulation	Moderate; ICD induction + polysaccharide adjuvant effect (Poria cocos–AuNRs; porphyran-coated AuNRs) [17,52]	Moderate; polydopamine-based nanoplatforms can induce ICD and promote dendritic cell activation; polysaccharide matrix (e.g., alginate) further contributes to immunomodulation [10]

**Table 5:** Representative studies of polysaccharide-modified AuNR and PDA systems for NIR-responsive biomedical applications. (Abbreviations: AuNRs, gold nanorods; PDA, polydopamine; HA, hyaluronic acid; DOX, doxorubicin; PK, pharmacokinetics; NIR, near-infrared; PTT, photothermal therapy; β-CD, β-cyclodextrin. All entries are supported by the cited original studies.).

Polysaccharide	Carrier	NIR Condition	Model	Main Outcome	Key Limitation
*Poria cocos* polysaccharide [17]	AuNRs	NIR	Cancer cells; zebrafish	Photothermal + photodynamic effects; inhibited tumor growth/metastasis	Limited drug loading
*Belamcanda chinensis* polysaccharide [47]	AuNRs	NIR	HepG2, A549, MCF-7	Inhibited liver cancer cell proliferation	No in vivo tumor model
HA/hydroxyethyl chitosan [86]	AuNRs	808 nm, pH/NIR	MCF-7	pH/NIR dual-responsive DOX release; enhanced chemo-PTT	No in vivo PK
HA [26]	PDA nanoparticles (systemic)	NIR	*S. aureus*, *E. coli*	100% antibacterial activity under NIR; limited in dark	No in vivo model
*Bletilla striata* polysaccharide [18]	PDA hydrogel (local)	808 nm	Diabetic wound; *S. aureus*, *E. coli*	NIR-triggered CO_2_ release; alleviated hypoxia; antibacterial/antioxidant	Local application only
Chitosan/alginate [120]	PDA hydrogel (local)	808 nm	Osteoblast cells	Enhanced mechanical properties; promoted osteoblast adhesion/proliferation	No in vivo NIR evaluation
Chitosan/β-CD [115]	PDA coating (interface)	Not required	Blood oxygenation	Reduced hemolysis (0.02%); low protein adsorption; stable after 24 weeks	Not NIR-responsive; interface only

## Data Availability

No new data were created or analyzed in this study. Data sharing is not applicable to this article.
